# Pine‐fungal co‐invasion alters whole‐ecosystem properties of a native eucalypt forest

**DOI:** 10.1111/nph.70363

**Published:** 2025-07-07

**Authors:** Corinne R. Vietorisz, Jake A. Nash, J. Alexander Siggers, Elena J. Leander, Beatrice M. Bock, Lennel A. Camuy‐Vélez, Allie Jasmine Hall, Joseph E. Jaros, Kevin A. Kuehn, Edith Y. Lai, Ian R. Mounts, Ivory J. Bacy, Caitlin E. Dagg, Ian C. Anderson, Angus J. Carnegie, Jeff R. Powell, John Stephen Brewer, Carla M. D'Antonio, Nicole A. Hynson, Rytas J. Vilgalys, Jason D. Hoeksema

**Affiliations:** ^1^ Department of Biology Boston University 5 Cummington Mall Boston MA 02215 USA; ^2^ Department of Biology Duke University 130 Science Drive Durham NC 27708 USA; ^3^ Department of Biology and Graduate Degree Program in Ecology Colorado State University 251 W Pitkin St Fort Collins CO 80521 USA; ^4^ Department of Integrative Biology University of Texas Austin, 2515 Speedway Austin TX 78712 USA; ^5^ Department of Biological Sciences Northern Arizona University Flagstaff AZ 86005 USA; ^6^ Center for Adaptable Western Landscapes Northern Arizona University Flagstaff AZ 86005 USA; ^7^ Microbiological Sciences North Dakota State University 1523 Centennial Blvd Fargo ND 58102 USA; ^8^ Pacific Biosciences Research Center University of Hawaiʻi at Mānoa 1993 East‐West Rd Honolulu HI 96815 USA; ^9^ Department of Evolution, Ecology, and Organismal Biology The Ohio State University Columbus OH 43210 USA; ^10^ School of Biological, Environmental, and Earth Sciences University of Southern Mississippi 118 College Dr. #5018 Hattiesburg MS 39406 USA; ^11^ Department of Ecology & Evolutionary Biology University of California Santa Cruz, 130 McAllister Way Santa Cruz CA 95060 USA; ^12^ Department of Biology University of Mississippi P.O. Box 1848 Oxford MS 38677 USA; ^13^ Hawkesbury Institute for the Environment Western Sydney University Hawkesbury Campus, Science Road Richmond NSW 2753 Australia; ^14^ Hawkesbury Institute for the Environment Western Sydney University Penrith NSW 2751 Australia; ^15^ Forest Science Department of Primary Industries Parramatta NSW 2150 Australia; ^16^ Environmental Studies University of California 4312‐L Bren Hall Santa Barbara 93105 USA

**Keywords:** co‐invasion, ectomycorrhizal fungi, *Eucalyptus*, leaf litter, microbial communities, pine invasion, soil moisture, suilloid fungi

## Abstract

- Pine‐fungal co‐invasions into native ecosystems are increasingly prevalent across the southern hemisphere. In Australia, invasive pines slowly spread into native eucalypt forests, creating novel mixed forests. We sought to understand how pine‐fungal co‐invasions impact interconnected above‐ and belowground ecosystem characteristics.
- We sampled beneath mature *Pinus radiata* and *Eucalyptus racemosa* in a pine‐invaded eucalypt forest in New South Wales, Australia. We measured microbial community composition via amplicon sequencing of 16S, ITS2, and 18S rDNA regions, microbial metabolic activity via Biolog plate substrate utilization, and soil, leaf litter, and understory plant characteristics.
- Pines were associated with decreased topsoil moisture, increased pine litter, and decreased eucalypt litter total phosphorus content. Soils and roots beneath pines had distinct microbial community composition and activity relative to eucalypts, including decreased bacterial diversity, decreased microbial utilization of several C‐ and N‐rich substrates, and enrichment of pine‐associated ectomycorrhizae. Introduced suilloid fungi were abundant across both pine and eucalypt soils and roots. Many ecosystem impacts increased with pine size.
- Invasive pines and their ectomycorrhizae have significant impacts on eucalypt forest properties as they grow. Interconnected impacts at the scale of individual trees should be considered when managing invaded forests and predicting effects of pine invasions.

## Introduction

Invasions of introduced plants have transformed ecosystems on a global scale, with nearly 14 000 documented naturalized non‐native vascular plant species (Van Kleunen *et al*., 2019). Invasive plant species can lead to negative outcomes for ecosystems and people including biodiversity loss, alteration of nutrient cycling, loss of local livelihoods (Simberloff *et al*., 2013; Paini *et al*., 2016), and breakdown of ecosystem resilience to environmental stressors. Plant–fungal interactions have proven to be important determinants of plant invasion success, whereby plant fitness may be enhanced by fungal mutualists, facilitating the establishment and proliferation of an invasive plant species (Desprez‐Loustau *et al*., 2007; Dickie *et al*., 2017). In many cases, the associated, typically mycorrhizal, fungi are also invasive, leading to plant–fungal co‐invasions (Nuñez & Dickie, 2014; Thakur *et al*., 2019). Despite the known importance of mycorrhizal fungi in dictating ecosystem function (Powell & Rillig, 2018) and the increasing awareness of their role in woody plant invasions, the ecosystem impacts of plant–fungal co‐invasions remain poorly understood.

The co‐invasion of pines and their ectomycorrhizal symbionts provides an important opportunity for understanding the ecosystem consequences of plant–fungal co‐invasions (Hoeksema *et al*., 2020). Pine trees (*Pinus* spp.) are almost exclusively native to the Northern Hemisphere, but their introduction to the Southern Hemisphere by European settlers in the 1800s has resulted in non‐native pines currently covering > 5 million ha (Simberloff *et al*., 2010). Pines are now considered invasive in South Africa, Australia, New Zealand, and South America, establishing in a variety of ecosystems including native grasslands, *Nothofagus* forests, shrublands, and *Eucalyptus* forests (Higgins & Richardson, 1998; Simberloff *et al*., 2010; Nuñez *et al*., 2017). Pines form obligate associations with mutualistic ectomycorrhizal fungi (EMF) and these mutualists are essential for their establishment and spread in their invasive range (Richardson *et al*., 1994). Beyond their initial co‐introduction into pine plantations, EMF spores disperse beyond plantation edges and enable the establishment of pines in adjacent native species‐dominated ecosystems (Nuñez *et al*., 2009; Policelli *et al*., 2019). The success of invasive pines is often driven by a single group of pine‐specific EMF, suilloid fungi, such as *Suillus* and *Rhizopogon* spp. (Hayward *et al*., 2015; Policelli *et al*., 2019). Still, it is uncertain how these widespread pine–EMF co‐invasions impact native forest ecosystem characteristics such as soil chemistry, overall microbial community diversity and composition, soil microbial metabolic activity, and leaf litter traits (Williams & Wardle, 2005; Hoeksema *et al*., 2020; Policelli *et al*., 2022; Lofgren *et al*., 2024).

In Australia, invasive radiata pine (*Pinus radiata*) can grow orders of magnitude faster than neighboring native eucalypts in hybrid pine–eucalypt forests (Burdon & Chilvers, 1977; Chilvers & Burdon, 1983). Such hybrid forests are now widespread in many regions of Australia where they persist without a classic ‘invasion front’ (Calviño‐Cancela & van Etten, 2018). Generally, pines may modify nutrient cycling and soil properties by producing large amounts of recalcitrant litter (Williams & Wardle, 2007), which decomposes slower than eucalypt leaf litter (Crockford & Richardson, 2002) while also decreasing soil pH. Suilloid fungi typically associated with pine invasions can thrive in relatively low‐nutrient environments (Wallander & Nylund, 1992; Hatakeyama & Ohmasa, 2004; Lilleskov *et al*., 2019) and may provide an advantage to their host plants by mining nutrients from the soil, increasing nutrient availability for host uptake. Thus, pines and their EMF symbionts may reduce soil total nutrient availability and moisture content through their slow‐decomposing litter, nutrient‐mining EMF, and high demand for nitrogen (N), phosphorus (P), and water relative to native eucalypts (Le Maitre *et al*., 2002). If established pines and their EMF decrease soil moisture and nutrient content, microbial community composition and activity is predicted to shift, as microbial community structure and enzymatic activity are heavily influenced by soil moisture and nutrient content (Fierer & Jackson, 2006; Brockett *et al*., 2012; Preece *et al*., 2019). Invasive EMF associated with pines can also alter broader microbial communities (Mujic *et al*., 2023), which may consequently affect native eucalypt growth and survival. As invasive pines grow, their influence on ecosystem properties is also predicted to increase, which can be examined by testing for associations of tree size with soil and microbial properties.

Given the impacts pines could have on eucalypt forests, we sought to answer the question: How might pine invasion into an intact eucalypt forest alter above‐ and belowground ecosystem characteristics? By sampling under a size gradient of invasive *P. radiata* within a native *Eucalyptus racemosa* forest and comparing microbial, litter, soil, and understory plant characteristics with those beneath nearby *E. racemosa* trees (also varying in size), we tested the following hypotheses: (1) Because invasive pines and EMF may uptake more water and nutrients than native eucalypts, they will be associated with decreased soil moisture and soil and litter total nutrient content. (2) Because invasive pines are typically associated with pine‐specific EMF communities and may alter soil properties that influence microbial communities, soil and roots beneath pines will have distinct microbial activity and communities, including more non‐native microbes, compared with soil and roots beneath eucalypts. (3) Changes in microbial community composition and diversity beneath invasive pines will be related to changes in litter inputs and soil metabolic activity, moisture, and nutrient content because these are typically major factors that influence microbial community composition. (4) These hypothesized differences in ecosystem characteristics between pines and eucalypts (H1–H3) will increase as tree size increases.

## Materials and Methods

### Study site characteristics

Samples were collected in Belanglo State Forest in New South Wales (NSW), Australia (34°31′18″S, 150°15′20″E; Supporting Information Fig. S1), in a woodland dominated by *E. racemosa* Cav., with the oldest individuals > 50 yr old. *Pinus radiata* D.Don have established in the woodland in a scattered pattern from a nearby plantation established in the 1960s. Hence, the oldest pines at the site were likely up to 50 yr old.

The Plant Community Type (PCT) for the site is Southern Highlands Scribbly Gum Forest, a mid–high to tall, sclerophyll open forest or woodland found on exposed aspects and shallow soils on Permo‐Triassic sandstone in the Southern Highlands. The tree canopy includes a high cover of native eucalypts, primarily *E. racemosa* and occasionally with *Eucalyptus dives* Schauer or *Eucalyptus mannifera* Mudie. A sparse‐to‐dense mid‐stratum of native heath species includes *Banksia spinulosa* Sm., *Hakea dactyloides* (Gaertn.) Cav., *Isopogon anemonifolius* (Salisb.), and *Persoonia mollis* R.Br. The ground layer is characterized by a sparse to mid‐dense and patchy cover of native graminoids that include *Patersonia glabrata* R.Br., *Lomandra obliqua* (Thunb.) J.F.Macbr., and *Entolasia stricta* (R.Br.). This PCT primarily occurs on crests and gentle exposed slopes within a narrow elevation range of 600–730 meters above sea level and in moderate‐to‐wet rainfall zones (800–1220 mm per annum). It is most extensive in the southwestern parts of the Southern Highlands.

### Field Sampling

In May 2023, we selected 9 *P. radiata* and nine *E. racemosa* trees > 15 cm in diameter at breast height (DBH) within a natural *E. racemosa* stand < 200 m from an adjacent *P. radiata* plantation (Fig. S1). Pines and eucalypts were selected to represent a range of sizes based on DBH. Although we made no effort to avoid large eucalypt trees, we found no eucalypt individuals with a DBH > 49.3 cm at the study site. By contrast, two pines were substantially larger than other trees included in the study (70 and 84.2 cm). Thus, our sampling effort included a larger range of pine (15.5‐ to 84.2‐cm DBH) sizes than eucalypts (20‐ to 49.3‐cm DBH). The average and median eucalypt DBH were 40.45 and 45.75 cm, respectively, while the average and median pine DBH were 40.22 and 33.30 cm, respectively (Dataset S1 for DBH of each individual). We measured the DBH of each tree and the distance to and height of the nearest heterospecific tree > 15‐cm DBH. We avoided trees that had conspecifics within a 3 m radius, or large (≥ 15 cm DBH) heterospecifics within a 1 m radius, to reduce overlapping zones of influence (Fig. S1).

We established a pair of 40 × 40 cm plots at each tree, 1 m due south and 1 m due north, to collect litter, soil, and root samples, and to measure vegetation characteristics (*n* = 36 total plots). Although this plot size and number of replicates would not be sufficient for assessing understory plant community dynamics across a forest, our goal was to assess the zone of influence surrounding each individual tree (Beiler *et al*., 2010). Within each plot, we visually estimated the percent cover of vegetation, pine litter, and eucalypt litter. We estimated vascular plant richness by counting all morphologically unique species within each plot. We then collected surface litter until mineral soil was reached, with the exception of debris larger than *c*. 5 cm in diameter. We collected soil and root samples using a garden trowel (sterilized with 70% ethanol) to a depth of *c*. 10 cm, collecting up to a volume of *c*. 1000 cm^3^. We placed collected materials in cold storage overnight and processed them at the Hawkesbury Institute for the Environment soil laboratory in Richmond, NSW. We air‐dried soil and litter intended for chemical analyses in paper bags before processing. We sorted litter into one of five categories: eucalypt bark and leaves, pine needles, woody debris from any plant species, upper Oe/Oa layer, and ‘other’, which was largely dead ferns (minimal in most plots). Woody debris included pine bark, pinecones, and small branches or twigs from any plant species. Organic layer material was a mix of partially decomposed leaves, decomposing male cones, and tiny branches that could not be separated from surrounding matter effectively. We discarded all live material, which was minimal. We further dried the sorted litter in a drying oven at 70°C for a minimum of 48 h and then weighed samples of each category. Fully expanded leaves were collected from live *L. obliqua* plants in each plot for total C/N/P analysis, as an indicator of nutrient availability in the understory (e.g. Gégout *et al*., 2005; Pinto *et al*., 2016). This C3 graminoid species was selected as representative because it was the most frequently observed understory species present near the sampled trees, occurring within 1 m of each sampled tree (with the exception of one eucalypt). We dried *L. obliqua* leaves at 70°C before grinding them with a Wig‐L‐Bug amalgamator (Crescent Dental).

### Leaf, litter, and soil property measurements

We ground soil to a fine powder in a ball‐mill and dried it at 65°C for total C and N analysis, and litter was ground in a Wiley mill for subsequent analyses of ergosterol and C/N/P content. For each soil and litter sample, we conducted analysis on two samples per tree (i.e. one per North and South plot). To determine the C and N contents in plant litter and soil samples, we used a Costech 4010 elemental combustion analyzer. We measured the phosphorus contents of litter (45 mg) and air‐dried soil (250 mg) subsamples using a SEAL autoanalyzer 3 (molybdate‐ascorbic acid method) following combustion (500°C) and hot hydrochloric acid extraction of ground litter samples (Lodato *et al*., 2021). We used ergosterol as a proxy for living and recently dead fungal biomass in air‐dried soil and litter (Grant & West, 1986; Newell *et al*., 1987; Weete *et al*., 2010). Further details regarding ergosterol extraction from litter and soils are fiven in Methods S1. We calculated gravimetric soil moisture on an *c*. 30 g sample of soil by drying overnight at 105°C followed by measuring organic matter by loss on ignition at 600°C for 4 h (Hoogsteen *et al*., 2018). We analyzed samples of ground *L. obliqua* leaf material for total % C and N via combustion in a CN analyzer, and for K and P after nitric acid/hydrogen peroxide digestion and analysis using inductively coupled plasma‐atomic emission spectroscopy.

### Microbial functional traits (Biolog Ecoplates™)

We used Biolog EcoPlates™ (catalog no. 1506) and Biology Phenotype Microarray Nitrogen Utilization Assay (PM8) plates (catalog no. 12183) to profile potential microbial carbon (C) and N substrate consumption. EcoPlates each contained 31 unique carbon sources (although these varied in being more N‐rich or C‐rich) in triplicate, and PM8 plates contained 94 different two‐ and three‐amino peptides. We subsampled and pooled 3 g of soil from the North and South samples of each focal tree and added 4 g of the pooled soil material into 40 ml of nanopure water in 50‐ml Falcon tubes. Each new sample was then shaken on an orbital shaker for 30 min at 22°C and refrigerated at 4°C for 30 min to allow the substrate to settle. We diluted the supernatant with sterile water at a 1 : 100 ratio and pipetted 150 μl of the dilution into each well of an EcoPlate and a PM8 plate. We also included additional blanks of each plate using 150 μl of nanopore water in each well instead of the sample dilution mix. We incubated the plates at 22°C with readings every 24 h for 5 d on a BMG LabTech CLARIOstar microplate reader at OD_590_. We used optical density (i.e. absorbance) values from Day 5, with values from blank plates subtracted, as a measure of potential microbial substrate consumption for each substrate.

### Microbial community sequencing

We conducted microbial community metabarcoding sequencing of soils and roots from each tree. For soil samples, DNA was extracted from sieved soils from each North and South plot around each tree. For root samples, roots from both the North and South plots were pooled, and two subsets of roots were selected for grinding and DNA extraction (Methods S1). We proceeded with two soil and two root samples per individual tree. We targeted the genes 16S for bacteria and archaea, ITS2 for general fungi, and 18S for arbuscular mycorrhizal fungi (AMF) to provide measures of microbial diversity and community composition. Libraries were sequenced on an Illumina MiSeq, and data were processed in QIIME2 with modified workflows specifically tailored to each gene. Detailed PCR and bioinformatics methods, including microbial guild assignments, are described in Methods S1 and Dataset S1.

We manually verified taxonomic annotations of fungi of interest, including introduced EMF and other abundant operational taxonomic units (OTUs), using blast searches (Altschul *et al*., 1990) against the NCBI Core Nucleotide Database. We used a phylogenetic approach to identify potentially ectomycorrhizal *Sistotrema* spp. (Methods S1) because they have previously been identified as dominant invasive fungi in pine plantations in New Zealand and are highly polyphyletic, which necessitates close examination. We identified 12 introduced EMF OTUs from the northern hemisphere and 12 confirmed native Australian EMF OTUs by reviewing > 99% blast hits and confirming that they were from the region of interest (either northern hemisphere or Australia). For the introduced fungi, this included well‐described fungal introductions including *Suillus* spp., *Rhizopogon* spp., *Lactarius deliciosus* (L.) Gray, *Amanita muscaria* (L.) Lam., and *Thelephora terrestris* Ehrh., as well as additional taxa that we confirmed were introduced with high confidence (Hoeksema *et al*., 2020). The list of 12 native fungi included some undescribed species in the genera *Laccaria*, *Lactarius*, and *Tomentella*, which had multiple high‐identity blast matches to Australian fungal collections.

### Statistical analyses

We conducted all analyses in R v.4.3.2 (R Core Team, 2024). To identify overall differences in biotic and abiotic variables between eucalypts and pines, we analyzed 102 response variables, including leaf, litter, and soil properties, as well as microbial diversity indices (i.e. richness and Shannon diversity) and substrate utilization rates (Dataset S1). We calculated alpha diversity metrics for each taxonomic group (i.e. bacteria/archaea, fungi, and AMF), including richness (i.e. number of unique taxa per sample) and Shannon diversity using the diversity function from the vegan package (Oksanen *et al*., 2013), and then, we calculated Cohen's *d* to quantify the effect size of tree species (pine or eucalypt) on response variables using the escalc function from the metafor package. Variables differing significantly in this overall test were visualized as a forest plot using tidyr (Wickham *et al*., 2024).

To assess differences in soil and root bacterial, fungal, and AMF community composition between tree species, we calculated robust Aitchison distances and conducted permutational multivariate analysis of variance tests using the adonis2 function in the vegan package. For each test, we assessed the assumption of multivariate homogeneity of variance using the BetaDisper function in the vegan package. We constructed principal coordinates analysis (PCoA) from the robust Aitchison distances of each root/soil and amplicon combination using the vegdist and cmdscale functions. We subsequently visualized PCoA plots using functions from the ggplot2 package (Wickham, 2016).

To test the relationship between key environmental variables and microbial communities, we conducted distance‐based redundancy analysis (dbRDA) of robust Aitchison distances using the capscale function in vegan. Pearson's correlation coefficients were generated for each environmental variable using the rcorr function in *Hmisc* to assess significant correlations with each other before use in dbRDA (Harrell, 2025). Variables of interest that were not significantly correlated with each other (i.e. soil total N, soil total P, soil moisture, tree DBH, litter depth, proportion of pine litter, and litter biomass) were used as predictors in dbRDA analysis of soil bacterial, fungal, and AMF communities. We tested for effects of tree species on microbial genera by conducting Analysis of Compositions of Microbiomes with Bias Correction 2 (ANCOM‐BC2, Lin & Peddada, 2020) on the 16S, 18S, and ITS2 feature tables after summing taxon abundances at the genus level. To test for differences in abundance between pines and eucalypts among introduced and native taxa, we ran ANCOM‐BC2 on individual fungal OTUs. We conducted ANCOM‐BC‐2 tests separately for the soil and root samples. To examine how pine size impacts EMF communities, we ran ANCOM‐BC2 on all soil EMF OTUs between small (< 40‐cm DBH) and large pines (> 40‐cm DBH).

To assess the strength of relationships between taxa enriched under pine or eucalypts and environmental variables, we ran Pearson's correlations using the rcorr function in the hmisc package. For all environmental variables that significantly differed with tree species (as determined by Cohen's *d* described previously), correlations were estimated with bacterial Shannon diversity, the top five most enriched fungal genera under eucalypts and pines, and the top five most enriched bacterial genera under eucalypts and pines. We chose to examine the top five most enriched fungal and bacterial genera because they typically had a log fold change greater in magnitude than 1, which indicates strong enrichment under pines or eucalypts.

To assess how the relationships between environmental variables and tree species changed with tree DBH, we constructed linear models explaining each environmental variable with the main effects of tree species and DBH, as well as the interaction between tree species and DBH, using the lm function in the stats package (R Core Team, 2024). We then ran the ANOVA function in the car package (Fox & Weisberg, 2019) on the linear model output to test for significance of tree species, DBH, and their interaction, using Type II sums of squares. Linear model assumptions of homoscedasticity and normality were checked by examining plots of the residuals vs fitted values and normal quantile–quantile plots, respectively.

We ensured there was no or limited spatial autocorrelation in microbial community composition, microbial genera abundances, Biolog substrate activity, and soil and litter properties among samples as described in Methods S1. We also confirmed that latitude and longitude did not have an effect in models testing the effects of tree species and DBH on microbial variables as described in Methods S1. Based on the results shown in Dataset S1, no samples were removed due to spatial autocorrelation.

## Results

**Fig. 1:**
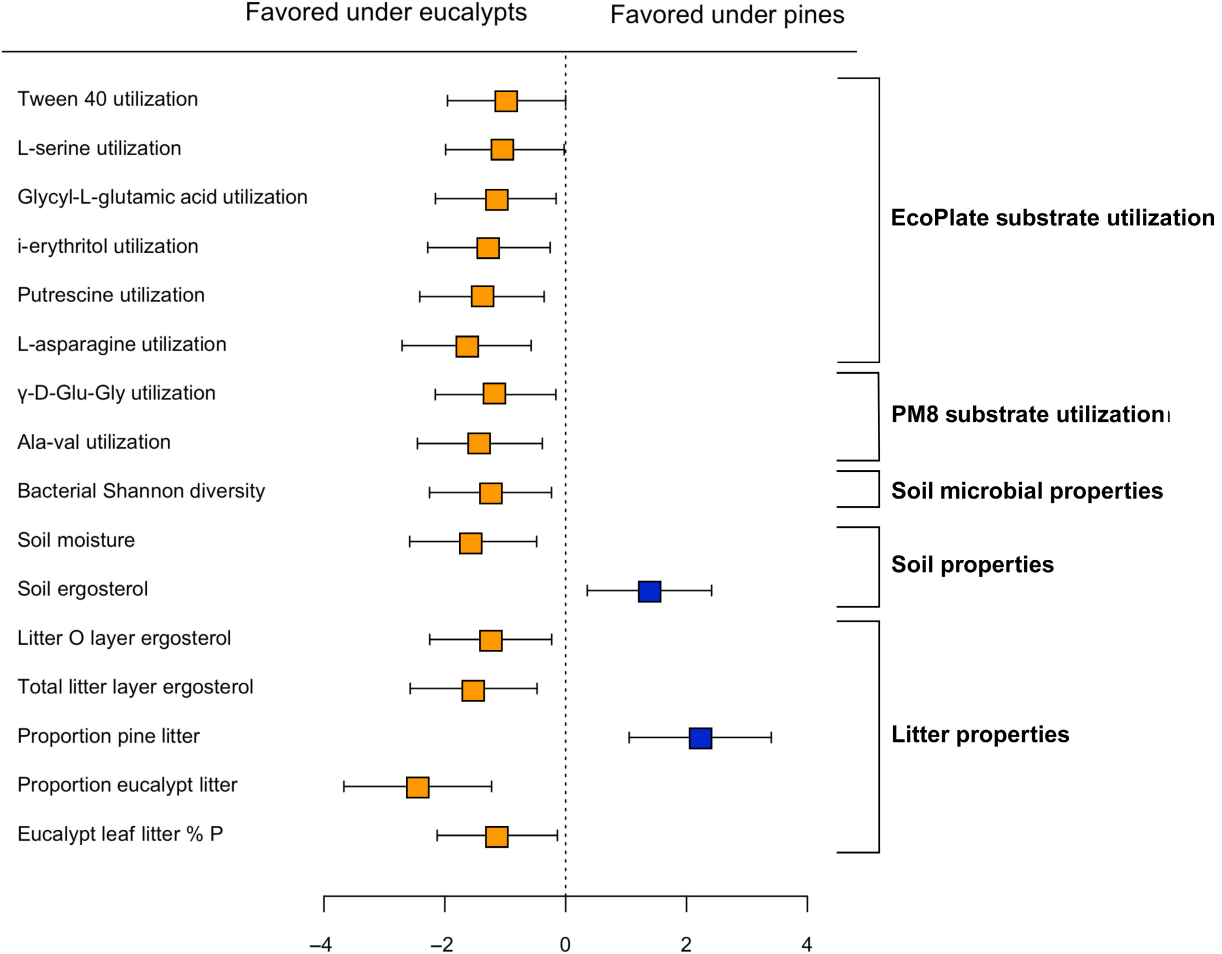
Forest plot illustrating the effect sizes (calculated using Cohen's *d*) for response variables that differ significantly under *Pinus radiata* (Pines) vs *Eucalyptus racemosa* (Eucalypts). Negative effect sizes indicate variables favored under *E. racemosa*, while positive effect sizes indicate variables favored under *P. radiata*. Error bars show 95% confidence intervals.

In partial support of our first hypothesis, pines were associated with decreased topsoil moisture, and eucalypt litter beneath pines had lower total P content (Fig. 1), but there were no differences in soil total nutrient content, soil % organic matter (loss on ignition), understory plant richness, or *L. obliqua* leaf nutrient content between pines and eucalypts (Figs S2, S3; Dataset S1). Yet, other soil and litter characteristics differed with tree species, including the proportions of pine and eucalypt leaf litter and fungal biomass in soil and leaf litter (i.e. ergosterol concentrations; Figs 1, S2, S3). Although pine or eucalypt leaf litter % C or N did not differ beneath the two tree species (Fig. S2), pine leaf litter had significantly lower %N than eucalypt leaf litter across all samples (Fig. S4). Soil ergosterol was higher under pines, while leaf litter O layer ergosterol was higher under eucalypts (Fig. 1).

**Fig. 2:**
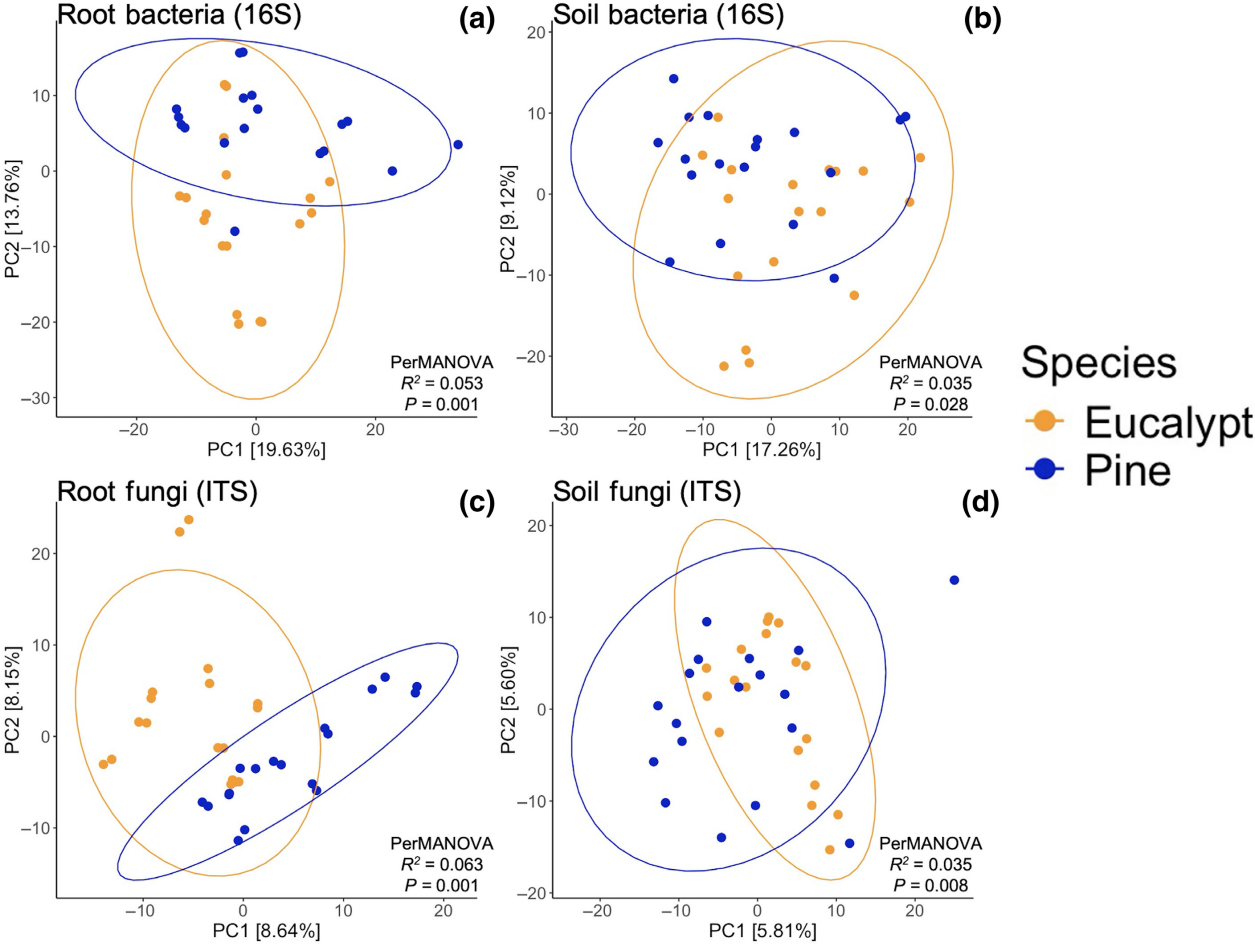
Principal coordinates analysis assessing differences in microbial community structure beneath *Pinus radiata* (Pine) vs *Eucalyptus racemosa* (Eucalypt) based on robust Aitchison distance. (a) Root bacterial (16S), (b) Soil bacterial (16S), (c) Root fungal (ITS2), (d) Soil fungal (ITS2). The first (PC1) and second (PC2) principal coordinate axes are shown for each sample type. *P. radiata* samples are colored blue and *E. racemosa* samples are colored yellow. Ellipses represent 95% confidence intervals. *R* ^2^ and *P*‐values were calculated using permutational multivariate analysis of variance tests.

**Fig. 3:**
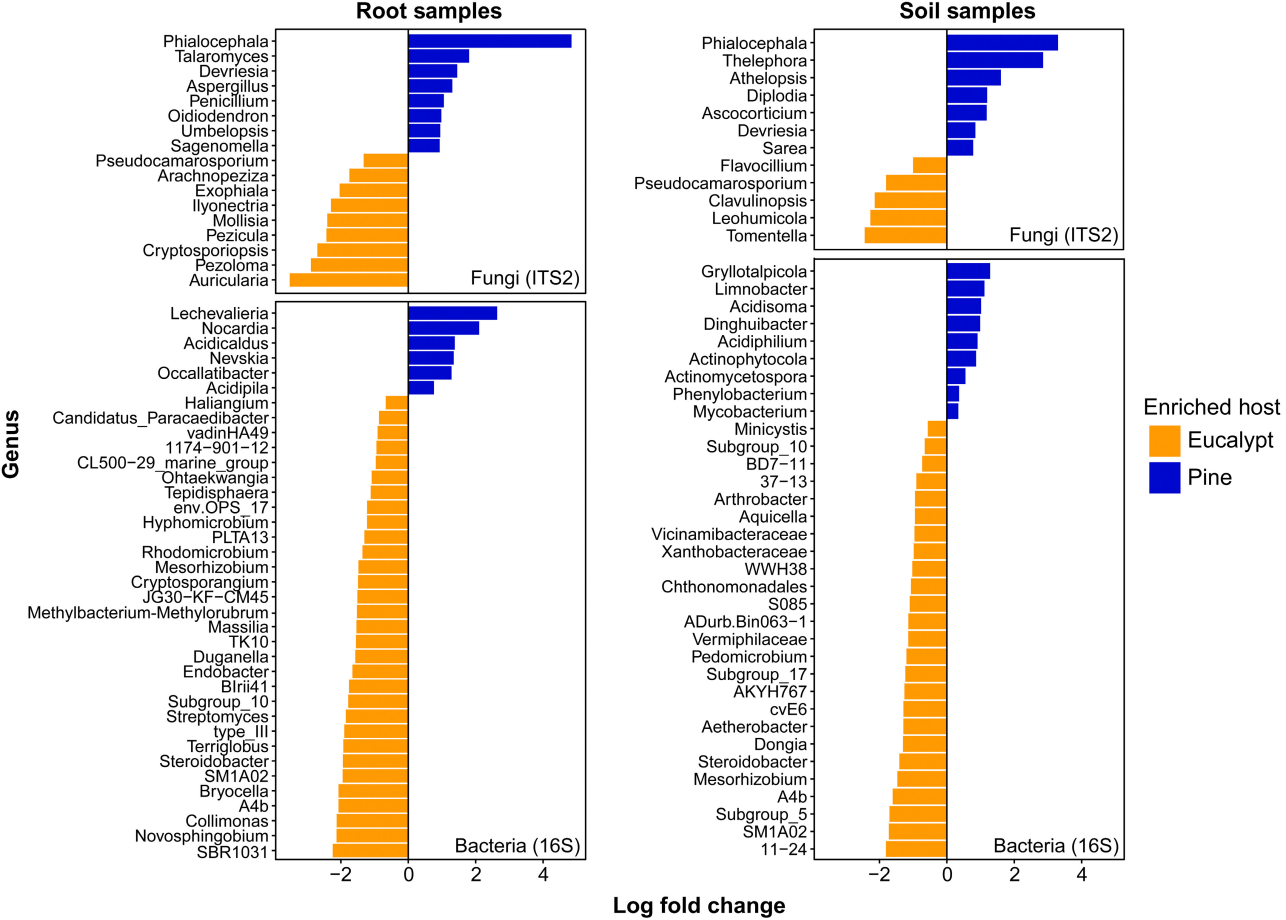
Results of ANCOM‐BC2 tests to detect fungal and bacterial genera that showed significant differential abundance between *Pinus radiata* (Pine) and *Eucalyptus racemosa* (Eucalypt), with separate tests performed for the root and soil datasets. Negative values of log_2_foldchange indicate higher sequence abundance on eucalypts, while positive values indicate higher sequence abundance on pines. Genera enriched under *P. radiata* are colored blue and genera enriched under *E. racemosa* are colored yellow. Only fungal genera that showed significant differences between hosts are displayed. There were no significantly enriched genera from the 18S arbuscular mycorrhizal fungi (AMF) dataset.

In support of our second hypothesis, soils and roots beneath pines had distinct microbial activity and community composition than those collected beneath eucalypts (Figs 1, 2). Microbial utilization of EcoPlate substrates Tween 40, l‐serine, glycyl‐l‐glutamic acid, i‐erythritol, and putrescine were all higher under eucalypts, as were utilization of amino acid peptides gamma‐d‐glutamylglycine (γ‐d‐Glu‐Gly) and the dipeptide alanine‐valine (Ala‐Val) (Fig. 1). Our metabarcoding approach successfully captured the vast majority of fungal, bacterial, and arbuscular diversity present in our samples based on rarefaction curves (Fig. S5). Pines and eucalypts had significantly different bacterial composition in roots (Fig. 2a; *R* ^2^ = 0.05, *P* < 0.001) and soils (Fig. 2b; *R* ^2^ = 0.03, *P* = 0.03), and eucalypt soil bacterial communities had greater betadispersion relative to pines (BetaDisper, *P* = 0.046). Fungal (ITS2 region) composition differed across pines and eucalypts in roots (Fig. 2c; *R* ^2^ = 0.06, *P* < 0.001) and soils (Fig. 2d; *R* ^2^ = 0.04, *P* = 0.008), while AMF (18S region) composition only differed in roots (Fig. S6; *R* ^2^ = 0.03, *P* = 0.045). Bacterial Shannon diversity was also significantly lower in pine soils than in eucalypt soils (Fig. 1). While fungal and bacterial functional guild abundances did not differ between tree species (specifically the relative abundances of ectomycorrhizal, arbuscular mycorrhizal, saprotrophic, and endophytic fungi, and copiotrophic and oligotrophic bacteria; Figs S7, S8), there were many differentially abundant fungal and bacterial genera between pines and eucalypts in both soils and roots (Fig. 3). Overall, more bacterial genera were differentially abundant in eucalypt soils and roots than in pine (Fig. 3). Many EMF genera were enriched under pines or eucalypts, including the introduced pine associates *Phialocephla* (in pine soils and roots) and *Thelephora* (in pine soils), while ectomycorrhizal *Tomentella* was the most enriched fungal genus in eucalypt soils (Fig. 3). There were five *Sistotrema* OTUs that had phylogenetic affinities suggesting an ectomycorrhizal lifestyle (Fig. S9) and that were exclusively found in eucalypt soils, although the genus *Sistotrema* was not differentially abundant beneath eucalypts. These OTUs are not closely related to the highly abundant introduced ectomycorrhizal *Sistotrema* OTUs found in a study of New Zealand pine plantations by Sapsford *et al*. (2022). There were no AMF genera enriched under pines or eucalypts.

**Fig. 4:**
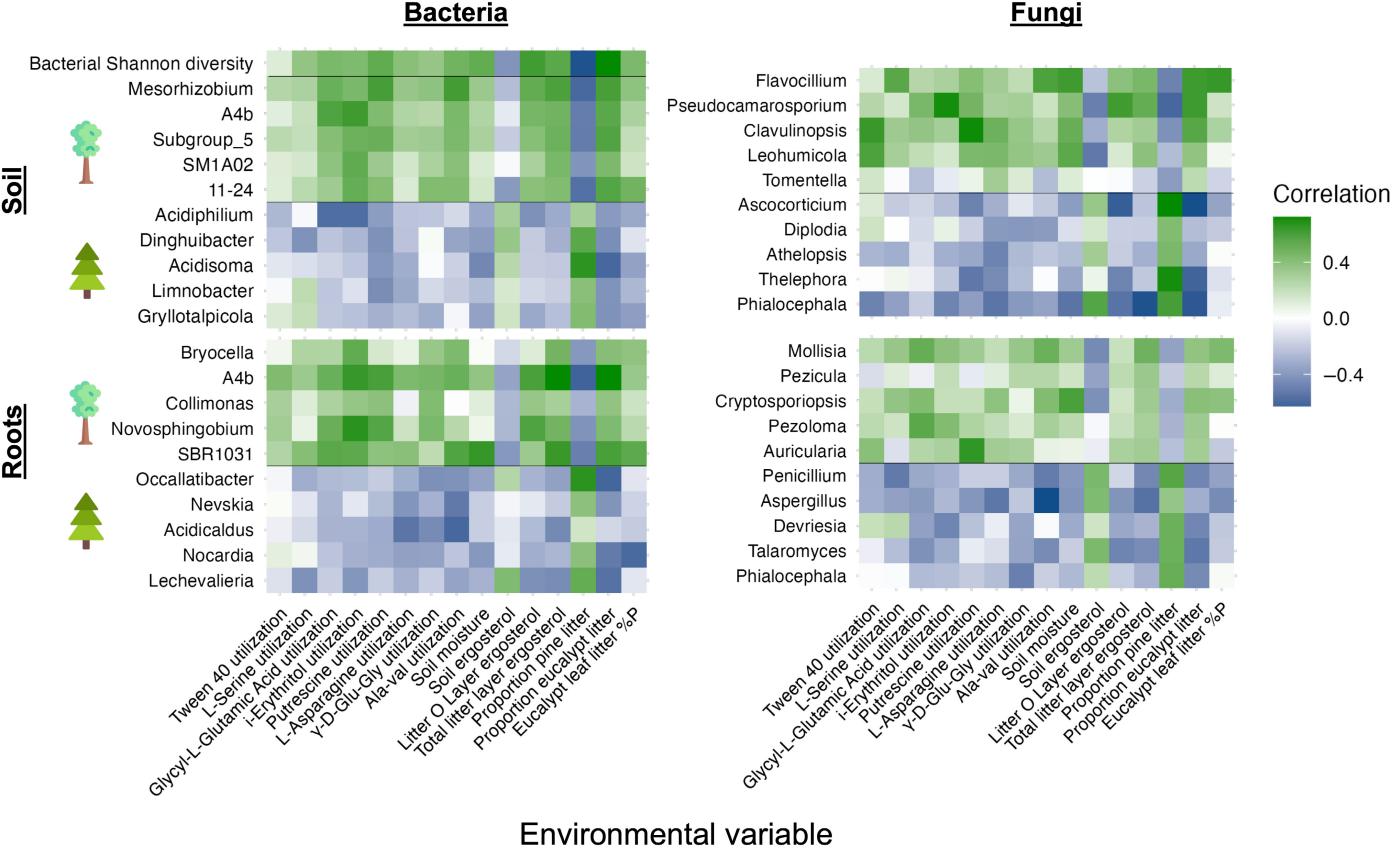
Pearson's correlations between microbial and environmental variables that differ between *Pinus radiata* and *Eucalyptus racemosa*. The most differentially abundant genera of soil bacteria, root bacteria, soil fungi, and root fungi are shown on the *y*‐axes of each heatmap. The top five differentially abundant genera in *E. racemosa* samples are listed on the top half of each heatmap, and the top five differentially abundant genera in *P. radiata* samples are listed on the bottom half of each heatmap. Bolded lines separate genera enriched in *E. racemosa* vs *P. radiata*, and bacterial genera from bacterial Shannon diversity.

Microbial factors that differed with tree species frequently correlated with the soil and litter characteristics that also differed under pines vs eucalypts, including soil metabolic activity, soil moisture, soil and litter fungal biomass, litter composition (i.e. proportion of pine or eucalypt litter), and litter total nutrient content (Fig. 4). Bacterial Shannon diversity correlated positively with many environmental variables, but most strongly with the soil metabolic activity of glycl‐l‐glutamic acid, i‐erythritol, putrescine, and Ala‐Val, soil moisture, litter ergosterol, eucalypt litter %P, and the proportion of eucalypt litter (Fig. 4). Bacterial genera enriched in eucalypt soils and roots had the strongest correlations with environmental variables, especially soil metabolic activity and litter characteristics (Fig. 4). Fungal genera enriched in roots correlated largely with litter characteristics, but fungal genera enriched in soils did not consistently correlate with any group of variables (Fig. 4).

**Fig. 5:**
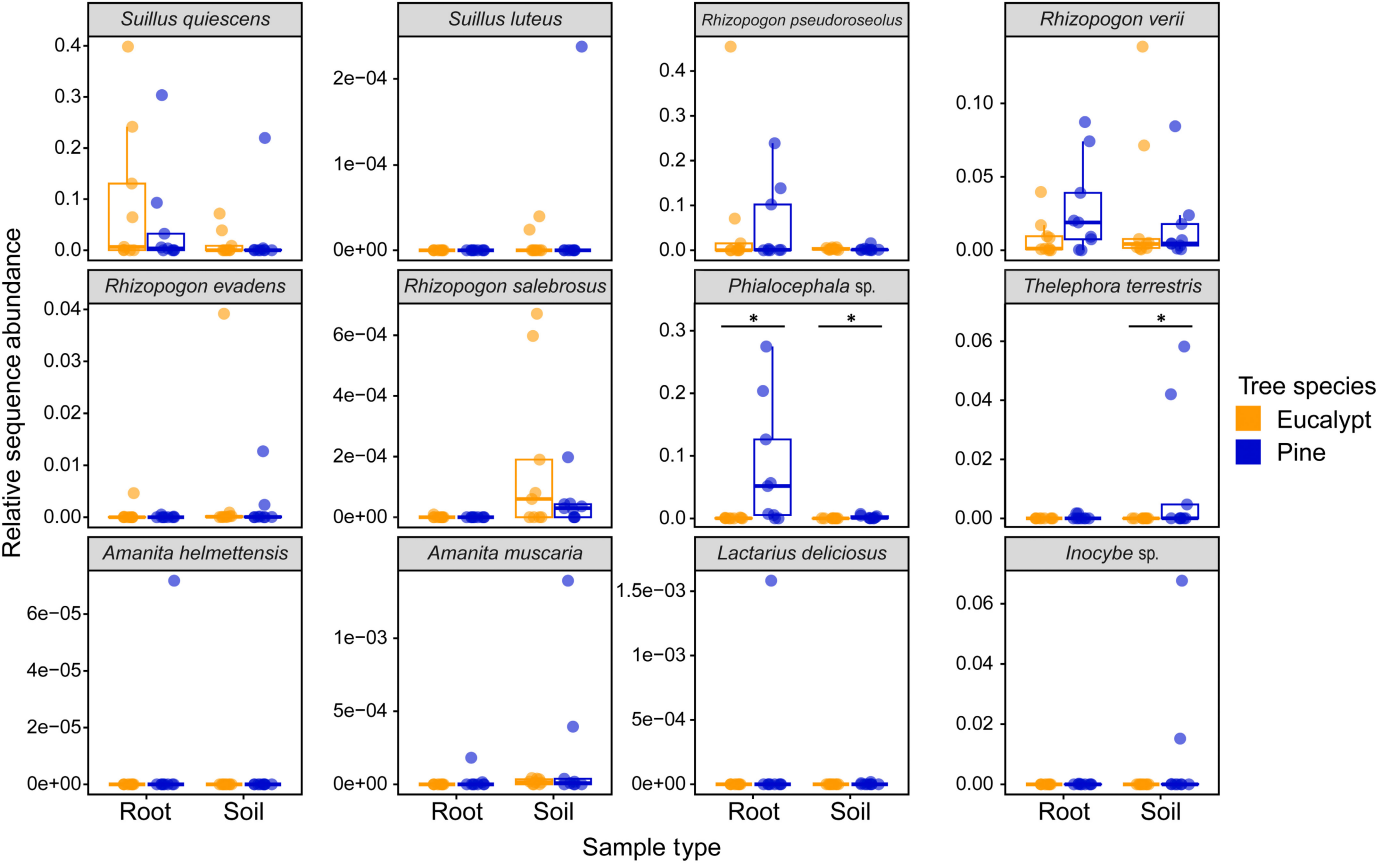
Occurrence patterns of 12 operational taxonomic units of introduced ectomycorrhizal fungi in roots and soil on pines and eucalypts. Boxes represent the interquartile range of the data, midpoint lines represent the median, whiskers represent data within 1.5 times the interquartile range, and points represent the values of each sample. Significance from ANCOM‐BC2 testing for differences between *Pinus radiata* (Pine) and *Eucalyptus racemosa* (Eucalypt) samples is indicated with an asterisk. *P. radiata* samples are colored blue and *E. racemosa* samples are colored yellow. Relative abundance is calculated as a proportion of the total sequences in the sample.

We found mixed support for our hypothesis that pine roots and soils host more non‐native fungi than nearby eucalypts. Introduced *Suillus* and *Rhizopogon* spp. were prevalent EMF community members in both pine and eucalypt roots and soils and did not show enrichment on either host (Figs 5, S10). However, we found that the introduced pine‐associated EMF *Phialocephala* sp. and *T. terrestris* showed strong association with pines, and little or no association with eucalypt roots or soils (Fig. 5). All 12 Australian native EMF OTUs were generally very low in abundance and occurred only sporadically across roots and soils (Fig. S11). Several native Australian taxa, including *Inocybe nudiuscula, I. subferruginea, Russula echidna, Laccaria* sp. 3, and *Tomentella* sp. 1, were found primarily with eucalypts, while other native EMF, such as *Tomentella* sp. 2, *Laccaria* sp. 1 and 2, *Lactarius* sp., *I. squamosa*, and *I. serrata* were observed in pine roots and/or soils (Fig. S12). When examining drivers of whole microbial community composition, the full bacterial (*F* = 1.1, *P* = 0.003) and fungal (*F* = 1.0, *P* = 0.045) dbRDA models, including soil total nitrogen, soil total phosphorus, soil moisture, tree DBH, litter depth, and proportion of pine litter, explained a significant amount of variation in community composition (42.5% and 42.03%, respectively). The first axis of the bacterial (Fig. S12a; *F* = 1.3, *P* = 0.007) and AMF (Fig. S12c; *F* = 1.3, *P* = 0.004) dbRDA models explained significant variation in community composition (7.53% and 7.49%, respectively). Across all dbRDAs, soil %N was a significant indicator of soil microbial community composition (Fig. S12; *P* < 0.01), even though soil %N did not differ beneath pines vs eucalypts. The proportion of pine litter was also a major influence on soil bacterial (Fig. S12a; *F* = 1.1, *P* = 0.02) and fungal (Fig. S12b; *F* = 1.1, *P* = 0.002) community composition, while tree DBH influenced soil AMF community composition (Fig. S12c; *F* = 1.1, *P* = 0.045).

**Fig. 6:**
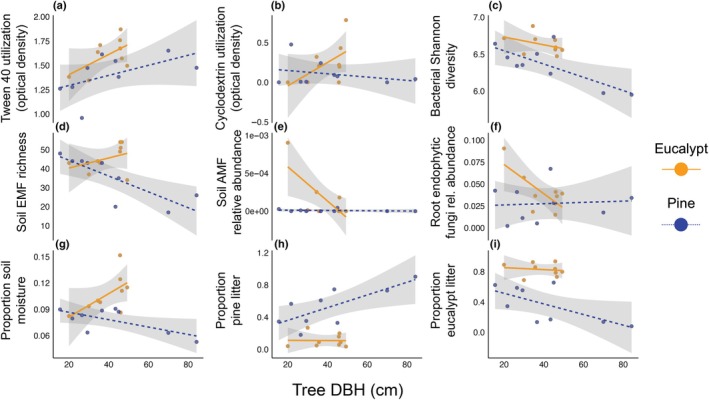
Linear regressions show the relationships between tree diameter at breast height (DBH) and (a) EcoPlate Tween 40 utilization, (b) EcoPlate Cyclodextrin utilization, (c) bacterial Shannon diversity, (d) soil ectomycorrhizal fungi (EMF) richness, (e) soil arbuscular mycorrhizal fungi (AMF) relative abundance, (f) root endophytic fungi relative abundance, (g) proportion topsoil moisture, (h) proportion of pine litter, and (i) proportion of eucalypt litter. Only response variables where both the main effects of tree species and DBH, or the interaction between tree species and DBH, were significant are shown. Significance was determined by running an ANOVA on the linear model output. Gray shading around each regression line shows the 95% confidence interval. *Pinus radiata* (Pine) samples are colored blue and *Eucalyptus racemosa* (Eucalypt) samples are colored yellow.

In partial support of our fourth hypothesis, some environmental differences between pines and eucalypts were exacerbated as tree size (DBH) increased (Fig. 6, Dataset S1). Some variables exhibited differing trends with tree size beneath pines vs eucalypts, with a significant interaction between tree species and DBH (Fig. 6b,d,g): cyclodextrin utilization (*F* = 4.9_1,14_, *P* = 0.04), soil EMF richness (*F* = 5.8_1,14_, *P* = 0.03), and soil moisture (*F* = 8.9_1,14_, *P* = 0.01) all increased with increasing eucalypt size but decreased with increasing pine size. There were seven EMF OTUs that significantly decreased in abundance with increasing pine size that may be partially responsible for the observed decline in EMF richness in response to tree DBH in pine soil, although the magnitude of the decline in richness cannot be explained solely by these seven OTUs. Root AMF (*F* = 14.6_1,14_, *P* = 0.002) and endophytic fungi (*F* = 5.0_1,14_, *P* = 0.04) relative abundances decreased with increasing eucalypt size but remained constant across pine sizes (Fig. 6e,f). While the interaction between tree species and DBH was not significant for other variables, the main effect of tree DBH significantly impacted several factors: The proportion of pine litter increased (*F* = 14.5_1,14_, *P* = 0.002), and the proportion of eucalypt litter decreased (*F* = 10.6_1,14_, *P* = 0.006), with increasing pine DBH (Fig. 6h,i). Other variables were related to tree DBH but exhibited similar trends between pines and eucalypts: Tween 40 utilization increased (*F* = 5.2_1,14_, *P* = 0.04) and bacterial Shannon diversity decreased (*F* = 15.1_1,14_, *P* = 0.002) with increasing DBH (Fig. 6a,c).

## Discussion

**Fig. 7:**
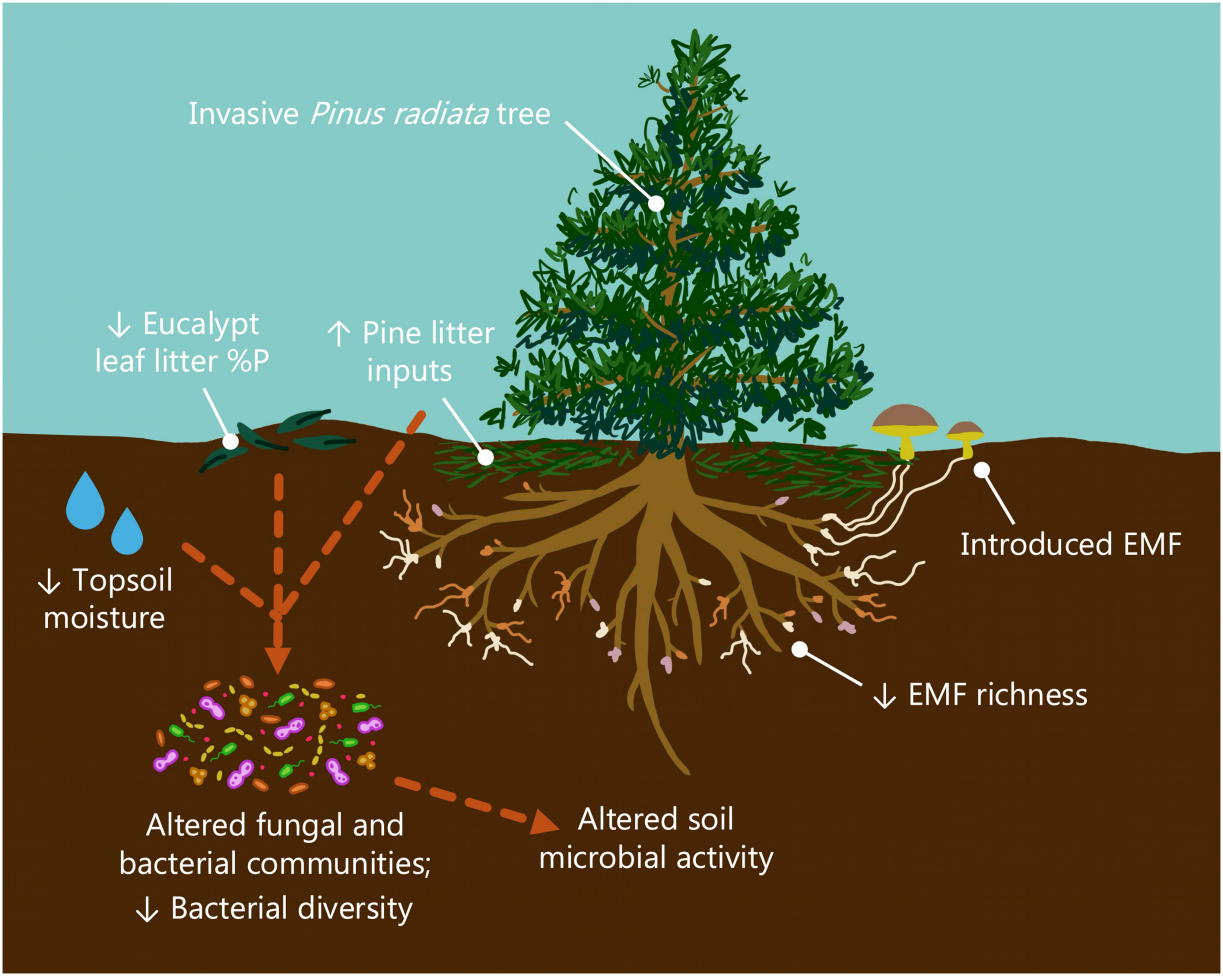
Conceptual diagram highlighting the ecosystem impacts of invasive *Pinus radiata* in a native eucalypt forest based on our results. Dashed arrows represent hypothesized connected impacts based on correlations between variables. Solid white lines indicate labels. Ectomycorrhizal fungi are abbreviated as ectomycorrhizal fungi (EMF). Illustration by Corinne Vietorisz.

We show that within an Australian mixed eucalypt–pine forest, pines were associated with unique ecosystem properties that differed from native eucalypts, and many of these differences increased with increasing pine size. As suggested by Burdon & Chilvers (1977), despite the coexistence of eucalypts with pines in this novel hybrid forest, invasive pines appear to be changing the native ecosystem over time. Many of these changes were interconnected (Fig. 7). As pines grew larger, they decreased topsoil moisture, increased pine litter inputs, and were associated with lower eucalypt leaf litter %P (Figs 1, 6g,h). Altered litter inputs and soil moisture may lead to distinct soil and root fungal and bacterial communities (Fig. 2), including decreased bacterial diversity (Figs 1, 6c). These distinct pine‐associated soil microbial communities had reduced utilization of several N‐ and C‐rich substrates compared with eucalypt‐associated microbial communities (Figs 1, 4). The dominant EMF in our study were introduced pine‐associated fungi. Additionally, there was a negative relationship between pine size and EMF richness and bacterial Shannon diversity, which suggests that effects of pine invasion on microbial communities will increase as pines grow larger (Fig. 6c,d).

Because the proportion of pine litter and soil moisture were strongly related to changes in soil microbial composition (Figs 4, S12), litter composition and soil moisture may be major factors controlling the abundances of key microbial taxa under pines vs eucalypts at our study site (Fig. 7), although host specificity may be a large factor controlling composition of EMF. As pines grow more quickly than native eucalypts (Burdon & Chilvers, 1977; Chilvers & Burdon, 1983), they may deplete topsoil moisture because of their high water requirements: Conifer plantations and invasions typically reduce streamflow and pose a great risk for reducing water availability in water‐limited systems (Bosch & Hewlett, 1982; Le Maitre *et al*., 2002; Swaffer & Holland, 2015). While we do not know the effects of pines on moisture in deeper soils in which trees may access water, decreased topsoil moisture can in turn decrease soil bacterial diversity and soil microbial activity (Orchard & Cook, 1983; Chen *et al*., 2007). The pines in our study also had lower leaf litter %N relative to eucalypts (Fig. S4) and contributed large amounts of pine needle litter. Lower nutrient pine litter inputs could shift soil microbial communities by producing carbon and nutrient substrates favorable to microbes specialized to decompose more recalcitrant litter (Bhatnagar *et al*., 2018; Feng *et al*., 2022). Differences in fungal biomass, indicated by ergosterol concentrations, suggested a shift in microbial functional groups and activity between the soil and litter layers of eucalypts relative to pines. The higher soil ergosterol under pines indicates greater fungal biomass in the soil, potentially due to a higher abundance of soil fungi, such as EMF, which typically increase in dominance during pine invasion (Sapsford *et al*., 2022). However, the higher litter ergosterol under eucalypts indicates greater fungal biomass in the litter layer, perhaps because eucalypt leaf litter is more labile than pine litter (Crockford & Richardson, 2002), leading to greater fungal colonization of leaf litter.

Biolog substrate utilization patterns may differ between pines and eucalypts because their microbial communities are adapted to different types of litter inputs. The higher degradation of several N‐rich substrates by eucalypt microbial communities, including l‐serine, l‐asparagine, glycyl‐l‐glutamic acid, alanine‐valine, and gamma‐d‐glutamylglycine (Brodowski *et al*., 2005; Moe, 2013; Fig. 1), may reflect the higher N content and faster N‐decomposition of eucalypt litter relative to *P. radiata* litter (Fig. S4; Crockford & Richardson, 2002), selecting for microbes that readily degrade labile N sources. Enhanced degradation of the C‐rich putrescine and i‐erythritol substrates may indicate higher abundances of microbes that can degrade labile C substrates in eucalypt soils (Su *et al*., 2024). Increased Tween 40 degradation, which can be a component in wax, may reflect the adaptations of these communities to inputs from the waxy coating of eucalypt leaves (Horn *et al*., 1964). The strong positive correlations between bacterial, but not fungal, genera enriched under eucalypts and Biolog substrate utilization, especially glycyl‐l‐glutamic acid, i‐erythritol, putrescine, Ala‐Val, and gamma‐d‐glutamylglycine (Fig. 4), suggest that the breakdown of these substrates may be due to primarily bacterial activity instead of fungal activity. Because the Biolog substrates we tested are largely decomposed by fast‐growing bacteria instead of fungi (Stefanowicz, 2006), we could not capture the extent of fungal metabolic activity at our site. Fungi may contribute more to the breakdown of recalcitrant substrates, for example through the use of oxidative and hydrolytic enzymes (Talbot *et al*., 2015). Because there was higher fungal biomass (ergosterol content) in soils beneath pines than eucalypts, pine‐associated soil microbial communities may have higher enzymatic activity targeting recalcitrant substrates not captured in the Biolog assays. However, a few fungal taxa correlated strongly positively with the utilization of some Biolog substrates and may contribute to their degradation, for example *Pseudocamarosporium* with i‐erythritol, *Auricularia* with putrescine, and *Mollisia* and *Pezoloma* with glycyl‐l‐glutamic acid (Fig. 4).

In addition to microbial activity, many bacterial and fungal taxa were differentially abundant between pines and eucalypts (Fig. 3). The two most enriched bacterial genera on pine roots and the most enriched genus in pine soils are within the phylum *Actinomycetota*, which have been shown to increase in bacterial communities undergoing stressful conditions and decreasing bacterial diversity (Jeon *et al*., 2024). Multiple bacterial genera enriched in eucalypt soils and roots are within the phyla *Acidobacteria* and *Chloroflexi*, which are associated with high C‐ and N‐decomposition capabilities (Belova *et al*., 2018; Kalam *et al*., 2020; Wang *et al*., 2023), which may explain their strong associations with the Biolog C and N substrate utilization (Fig. 4). Many of the saprotrophic fungal genera enriched under pines correlated strongly positively with the proportion of pine litter (Fig. 4), so these fungi may be specialized at decomposing pine litter, as saprotrophic fungi often have separate ecological niches based on litter chemistry (Bhatnagar *et al*., 2018). These fungal taxa may be producing enzymes that target recalcitrant substrates in pine litter that were not measured in the Biolog assays, explaining their lack of correlation with Biolog substrate utilization. Ectomycorrhizal *Tomentella*, which has been shown to associate with eucalypts even outside the native range of *Eucalyptus* (Sugiyama & Sato, 2021), was the most enriched genus in eucalypt soils, while the most enriched fungal genus in pine roots and soils was *Phialocephala* (Fig. 3). *Phialocephala* has been described as both a root endophyte and a ectomycorrhizal associate of trees in the *Pinaceae* family (Landolt *et al*., 2020; Stroheker *et al*., 2021). *Phialocephala* has been shown to form ectomycorrhizal structures and enhance nutrient uptake in pines (Jumpponen *et al*., 1998; Otgonsuren & Lee, 2012).

Many introduced EMF taxa that were not found in eucalypt samples are known to frequently or preferentially associate with pines, including *Phialocephala* sp., two *Amanita* species (Dyshko *et al*., 2024), and *T. terrestris* (Marx & Bryan, 1970; Fig. 5). For native Australian OTUs, strong conclusions regarding their host specificity are limited by the sporadic occurrences of these fungi. Additionally, there were many other EMF detected in our dataset that are possibly native to Australia, but we were unable to make a high‐confidence determination of provenance because many Australian fungal species are still undescribed (see the Materials and Methods section). In contrast to other pine‐specific introduced fungi, suilloid fungi (*Suillus* and *Rhizopogon* spp.) were found in both pine and eucalypt root and soil samples (Fig. 5) and were not differentially abundant between pines and eucalypts (Fig. 3), indicating that these widespread invasive fungi were capable of establishing on or near eucalypt trees adjacent to invasive pines. However, we do not know whether these EMF formed functional and morphological mycorrhizal associations with the eucalypts. Past studies have shown that *Suillus* can colonize some angiosperm hosts with or without morphological development of ectomycorrhizae (Lofgren *et al*., 2018; Liao *et al*., 2021). Potential compatibility of *Suillus* spp. with eucalypts as ectomycorrhizal symbionts or incidental hyphal colonizers is unexplored (Schneider‐Maunoury *et al*., 2020). Our findings show that invasive suilloid fungi can spread throughout an entire pine‐invaded eucalypt stand and are not limited to the areas immediately surrounding pines. Given that our study site borders a *P. radiata* plantation, large amounts of suilloid fungi inoculum may have spread across the native eucalypt forest from both the adjacent pine plantation and the localized invasive pines. Suilloid fungi are often associated with low‐diversity EMF communities (Collier & Bidartondo, 2009; Nuñez *et al*., 2009; Hayward *et al*., 2015; Policelli *et al*., 2019; Thompson *et al*., 2022), which is in line with our findings that soils beneath large pines had reduced EMF richness (Fig. 7). Some early‐colonizing EMF may preferentially establish on small pines, for example the seven OTUs we found to be significantly more abundant on small pines. However, EMF richness decreased by much > 7 OTUs on large pines, indicating that an additional random assortment of EMF OTUs are lost as invasive pines grow. It is possible that invasive and/or pine‐specific EMF outcompete other EMF for space on roots as the tree matures, as priority effects can determine the competitive ability of suilloid fungi (Kennedy *et al*., 2009).

Overall, fungal taxa that were enriched under pines or eucalypts did not correlate strongly with environmental variables that differed with tree species (Fig. 4). *Phialocephala, Tomentella*, and *Thelephora* did not correlate strongly with most environmental variables, suggesting that these EMF may be largely responding to tree host preferences rather than associated environmental conditions (Policelli *et al*., 2020). By contrast, bacterial genera enriched under eucalypts had stronger and more uniform correlations with environmental variables. For example, the relative abundances of many bacterial genera correlated strongly with soil moisture and litter species composition and nutrient content (Fig. 4), which are known to strongly influence bacterial community composition (Brockett *et al*., 2012; Evans *et al*., 2014; Ma *et al*., 2015; Urbanová *et al*., 2015; Yan *et al*., 2018). Thus, changes in soil moisture and litter inputs beneath pines may lead to shifts in bacterial community composition and genera abundances. It was surprising that soil and plant litter % C, N, or P did not differ beneath pines and eucalypts (Figs S2, S3) given that pines input large amounts of pine litter, which had lower %N than eucalypt leaf litter (Fig. S4). These results indicate that litter total nutrient content may not be strongly coupled to soil total nutrient content in these soils, or else is controlled by other factors such as understory plant nutrient uptake (Rolo *et al*., 2012). Pines could also affect the inorganic or mineral fractions of soil N and P without significantly changing the total nutrient content of the soil, for example through production of pine‐associated ectomycorrhizal exoenzymes that liberate inorganic nutrients from organic matter in soil (Talbot *et al*., 2015). The total C, N, and P content of soils may remain constant under pine invasion; however, soil total C and nutrients in pine‐invaded eucalypt stands could differ from uninvaded eucalypt stands, which was not measured in this study. Thus, either pine invasion did not change total soil C and nutrients at our site, or invasive pines altered soil total C and nutrients at the whole‐stand level such that there were no differences in soil total C and nutrients between tree species in the same stand. To gain a complete picture of how invasive pines alter C and nutrient cycling in eucalypt forests, future work should measure total, inorganic, and mineral fractions of C and nutrients within soils and plant litter and at larger spatial scales, including uninvaded eucalypt stands.

Many of the differences between pines and eucalypts were related to tree size (Fig. 6), especially for pines, suggesting that as pines grow larger in size, their impacts on native eucalypt forests increase in magnitude. The largest pines we sampled were substantially larger than the largest eucalypts we sampled, even though the native eucalypt forest pre‐dates the pine plantation at this site, suggesting that these invasive pines may grow faster and larger than native eucalypts and thus have disproportionate impacts on the native forest. The impact of an invasive species is conceptualized as the magnitude of each individual's effect on the ecosystem times the species' abundance (Parker *et al*., 1999; Sofaer *et al*., 2018). In this instance, the impact of invasive pines increases not only with their numerical abundance (i.e. number of pines in a eucalypt forest) but also with their size. Thus, both the number and size of invasive pines should be considered when predicting the impacts of pine invasion and targeting pine individuals for removal. For impacts that were dependent on tree size, these effects may be localized beneath or near individual pines. Pine invasion typically homogenizes ecosystems above‐ and belowground, especially through reducing plant and fungal diversity (García *et al*., 2018). While we found reduced bacterial and EMF diversity, reduced bacterial community beta dispersion, and fewer differentially abundant bacterial genera beneath pines, these effects were localized beneath pines and were not spread across the entire forest. Therefore, pines may homogenize belowground microbial communities close to the tree, but not beneath nearby native trees. However, some effects of pine presence may already be widespread and homogenous within the forest, for example the presence of co‐invasive EMF (Fig. 4). To understand which effects are widespread throughout invaded forests compared with uninvaded native forests, further work comparing above‐ and belowground ecosystem properties in uninvaded and invaded native forests is needed.

### Conclusions

We demonstrate that invasive pines in a native eucalypt forest are associated with distinct soil properties, litter inputs, microbial communities, and microbial activity relative to native eucalypts, and many of these impacts increased with pine size (Fig. 7). Although these invasions create novel mixed forests lacking the classic invasion fronts seen in grassland or arbuscular mycorrhizal (AM)‐dominated systems (Burdon & Chilvers, 1977; Chilvers & Burdon, 1983), in which pine invasions introduce EMF into a community in which they were previously absent (Sapsford *et al*., 2022), we still saw shifts in microbial communities and ecosystem properties beneath individual pine trees. Decreases in soil bacterial and EMF diversity, microbial utilization of several C‐ and N‐rich substrates, and soil moisture beneath large pines have the potential to negatively impact native eucalypt forests as pines grow larger, as diverse microbiomes are critical for native ecosystem functions (Baldrian, 2017; Lladó *et al*., 2017). Maintaining soil moisture is important for microbial and plant health as drought frequency and severity is expected to increase globally, particularly in southeastern Australia, with climate change (Van Dijk *et al*., 2013; Hoque *et al*., 2021). Because these impacts increased with pine size, invasion mitigation efforts may be more effective when targeting larger trees. Overall, our results suggest that invasive pines can have localized effects even within heterogeneous forests, and their interconnected above‐ and belowground impacts should be considered when managing and predicting the effects of pine–EMF co‐invasions.

## Competing interests

None declared.

## Author contributions

JDH, JSB, RJV, NAH and CMD conceptualized the idea for the project. JDH, JSB, RJV, NAH, CMD, AJC, ICA and JRP supervised the project. CRV, JAN, JAS, BMB, LAC‐V, AJC, AJH, JEJ, EYL, EJL, IRM, IJB, JSB, CMD, NAH, RJV and JDH created the sampling design, collected samples and conducted field measurements. CRV, JAN, JAS, BMB, LAC‐V, AJH, JEJ, KAK, EYL, EJL, IRM, IJB, JSB, CMD, NAH and JDH processed samples. JAN, JAS, CRV, BMB, LAC‐V, CED, AJH, JEJ, EYL, EJL and JSB analyzed the data. CRV, JAS, JAN, IRM, EYL, LAC‐V, EJL and AJH wrote the original manuscript. All authors contributed to edits and review of the manuscript. CRV, JAN and JAS contributed equally to this work as co‐first authors. JSB, CMD, NAH, RJV and JDH contributed equally to this work as co‐last authors.

## Disclaimer

The New Phytologist Foundation remains neutral with regard to jurisdictional claims in maps and in any institutional affiliations.

## Supporting information


**Dataset S1** An Excel Workbook containing metabarcoding PCR cycles, microbial taxonomy, microbial feature tables, diameter at breast height regressions, spatial autocorrelation results, and top microbial taxa.

**Fig. S1** Satellite images of the sampling site.
**Fig. S2** Plot of the effect size of tree species on litter variables.
**Fig. S3** Plot of the effect size of tree species on soil properties.
**Fig. S4** Percent nitrogen in pine and eucalypt leaf litter.
**Fig. S5** Rarefaction curves for the Interally Transcribed Spacer Region (ITS), 16S, and 18S datasets.
**Fig. S6** Principal coordinate analysis of arbuscular mycorrhizal fungi community structure.
**Fig. S7** Plot of the effect size of tree species on soil microbial properties.
**Fig. S8** Plot of the effect size of tree species on root microbial properties.
**Fig. S9** Phylogenetic tree of *Cantharellales* operational taxonomic units.
**Fig. S10** Relative abundances of select ectomycorrhizal fungi and other guilds.
**Fig. S11** Relative abundances of 12 native Australian ectomycorrhizal fungi.
**Fig. S12** Distance‐based redundancy analysis of soil microbial communities.
**Methods S1** Methods description for ergosterol measurement, metabarcoding, bioinformatics, and spatial autocorrelation analysis.
Please note: Wiley is not responsible for the content or functionality of any Supporting Information supplied by the authors. Any queries (other than missing material) should be directed to the *New Phytologist* Central Office.

## Data Availability

The analyses that were used to generate findings of this study are publicly available in the repository ‘Aus‐Invasions‐2023‐Course’ at https://github.com/jakenash12/Aus‐Invasions‐2023‐Course. Representative sequences for the ITS2, 16S, and 18S datasets are publicly available associated with the NCBI BioProject: accession no. PRJNA1259539.
