# Supplementary material for: Pine‐fungal co‐invasion alters whole‐ecosystem properties of a native eucalypt forest
Source: New Phytol. 2025 Jul 7;247(5):2342–56. doi: 10.1111/nph.70363 (PMC12329200; doi:10.1111/nph.70363)
Supplement: Supplementary file 2 — Fig. S1 Satellite images of the sampling site. Fig. S2 Plot of the effect size of tree species on litter variables. Fig. S3 Plot of the effect size of tree species on soil properties. Fig. S4 Percent nitrogen in pine and eucalypt leaf litter. Fig. S5 Rarefaction curves for the Interally Transcribed Spacer Region (ITS), 16S, and 18S datasets. Fig. S6 Principal coordinate analysis of arbuscular mycorrhizal fungi community structure. Fig. S7 Plot of the effect size of tree species on soil microbial properties. Fig. S8 Plot of the effect size of tree species on root microbial properties. Fig. S9 Phylogenetic tree of Cantharellales operational taxonomic units. Fig. S10 Relative abundances of select ectomycorrhizal fungi and other guilds. Fig. S11 Relative abundances of 12 native Australian ectomycorrhizal fungi. Fig. S12 Distance‐based redundancy analysis of soil microbial communities. Methods S1 Methods description for ergosterol measurement, metabarcoding, bioinformatics, and spatial autocorrelation analysis. Please note: Wiley is not responsible for the content or functionality of any Supporting Information supplied by the authors. Any queries (other than missing material) should be directed to the New Phytologist Central Office. [file NPH-247-2342-s001.pdf]

New Phytologist

***New Phytologist* Supporting Information**

Article title: Pine-fungal co-invasion alters whole-ecosystem properties of a native eucalypt forest
Authors: Corinne R. Vietorisz*, Jake A. Nash*, J. Alexander Siggers*, Elena J. Leander, Beatrice M. Bock, Lennel A. Camuy-Vélez, Allie Jasmine Hall, Joseph E. Jaros, Kevin A. Kuehn, Edith Y. Lai, Ian R. Mounts, Ivory J. Bacy, Caitlin E. Dagg, Ian C. Anderson, Angus J. Carnegie, Jeff R. Powell, John Stephen Brewer, Carla M. D'Antonio, Nicole A. Hynson, Rytas J. Vilgalys, Jason D. Hoeksema
Article acceptance date: 24 May 2025
*Corresponding authors: Corinne R. Vietorisz, Jake A. Nash, J. Alexander Siggers

The following Supporting Information is available for this article:

Fig. S1 Satellite images of the sampling site

Fig. S2 Plot of the effect size of tree species on litter variables

Fig. S3 Plot of the effect size of tree species on soil properties

Fig. S4 Percent nitrogen in pine and eucalypt leaf litter

Fig. S5 Rarefaction curves for the ITS, 16S, and 18S datasets

Fig. S6 PCoA of AMF community structure

Fig. S7 Plot of the effect size of tree species on soil microbial properties

Fig. S8 Plot of the effect size of tree species on root microbial properties

Fig. S9 A phylogenetic tree of *Cantharellales* OTUs

Fig. S10 Relative abundances of select ECM fungi and other guilds

Fig. S11 Relative abundances of 12 native Australian ECM fungi

Fig. S12 Distance based redundancy analysis of soil microbial communities

Methods S1 Methods description for ergosterol measurement, metabarcoding, bioinformatics, and spatial autocorrelation analysis

Dataset S1 An Excel Workbook containing metabarcoding PCR cycles, microbial taxonomy, microbial feature tables, DBH regressions, spatial autocorrelation results, and top microbial taxa

Fig. S1 Geographic locations of sampled pines and eucalypts. Sample IDs beginning with "E" indicate *Eucalyptus racemosa* individuals, and sample IDs starting with "P" indicate *Pinus radiata*. Ellipse size indicates tree size measured by diameter at breast (DBH) in centimeters, where orange is associated with eucalypt and blue with pine. Image generated using Airbus™ satellite imagery.

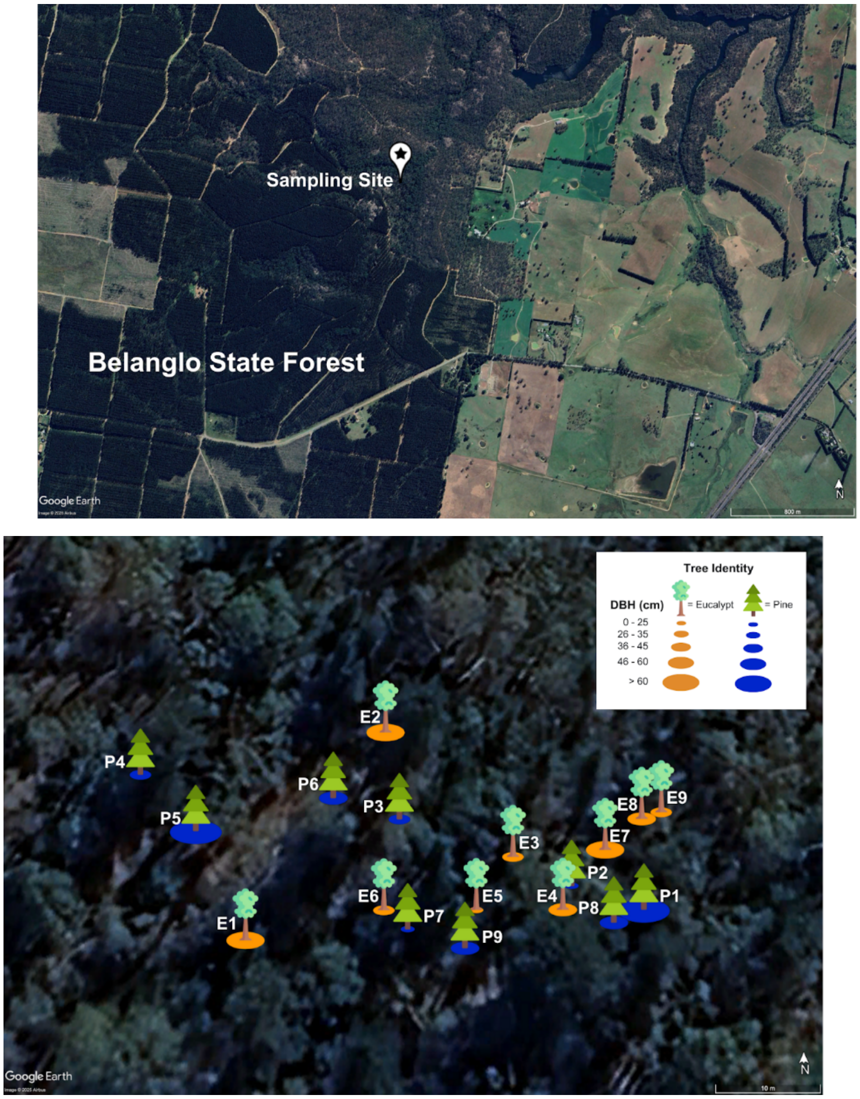

Fig. S2 Forest plot illustrating Cohen's *d* effect sizes for litter property response variables associated with *Pinus radiata* (Pines) versus *Eucalyptus racemosa* (Eucalypts). Negative effect sizes indicate variables favored under eucalypts, while positive effect sizes indicate variables favored under pines. Error bars show 95% confidence intervals.

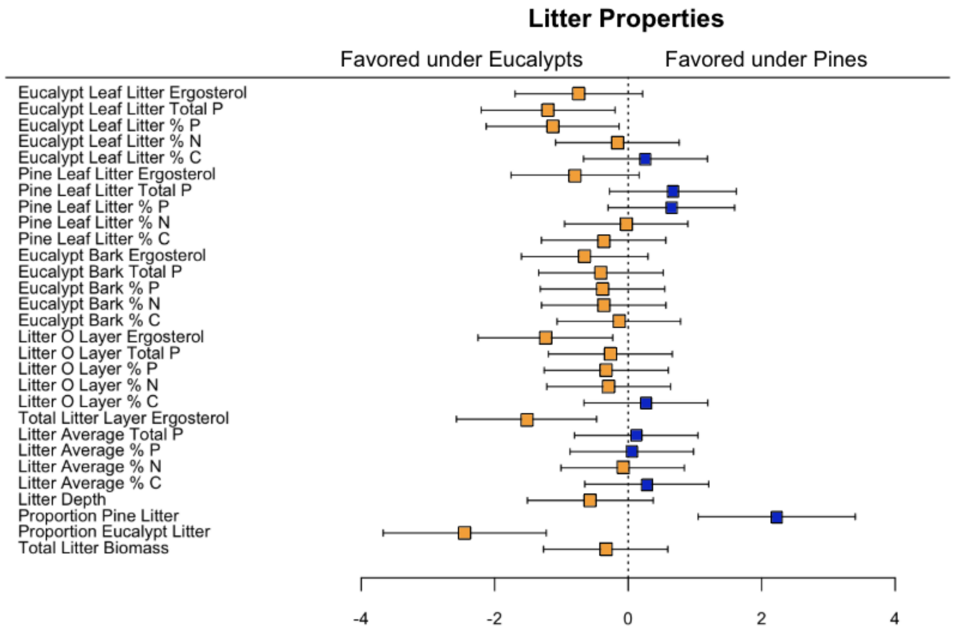

Fig. S3 Forest plot illustrating Cohen's *d* effect sizes for soil property response variables associated with *Pinus radiata* (Pines) versus *Eucalyptus racemosa* (Eucalypts). Negative effect sizes indicate variables favored under eucalypts, while positive effect sizes indicate variables favored under pines. Error bars show 95% confidence intervals.

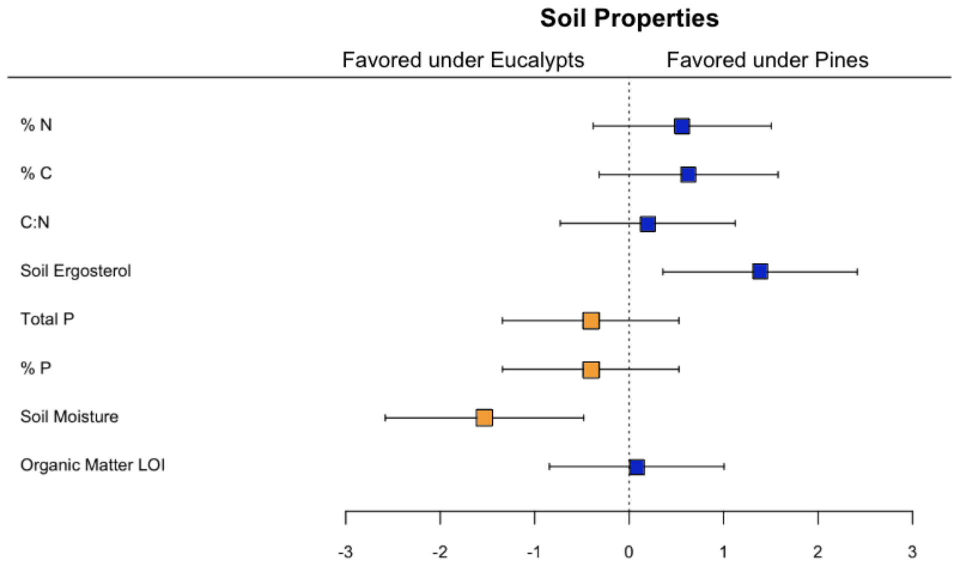

Fig. S4 Percent nitrogen in pine and eucalypt leaf litter across all samples. P-value was determined using an Anova test. The box represents the interquartile range of the data, the midpoint line represents the median, the whiskers represent data within 1.5 times the interquartile range, and points represent the values of each sample.

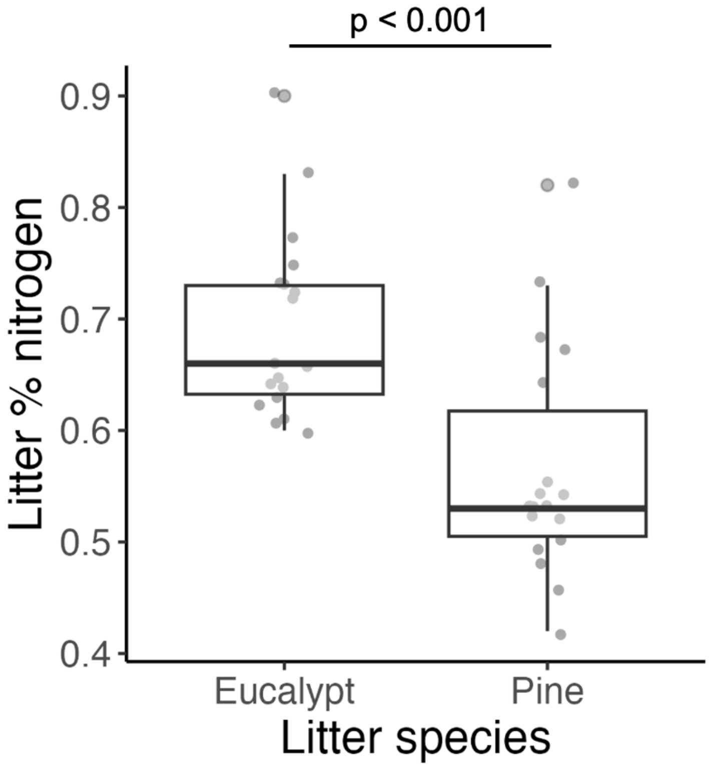

Fig. S5 Rarefaction curves for ITS, 18S, and 16S indicating the relation between sequencing depth and taxon richness for each sample. Each curve represents a unique sample and is color coded by tree species (*Pinus radiata* or *Eucalyptus racemosa*) and sample type (roots or soil). Dashed lines indicate the rarefaction subsampling depth chosen for each dataset.

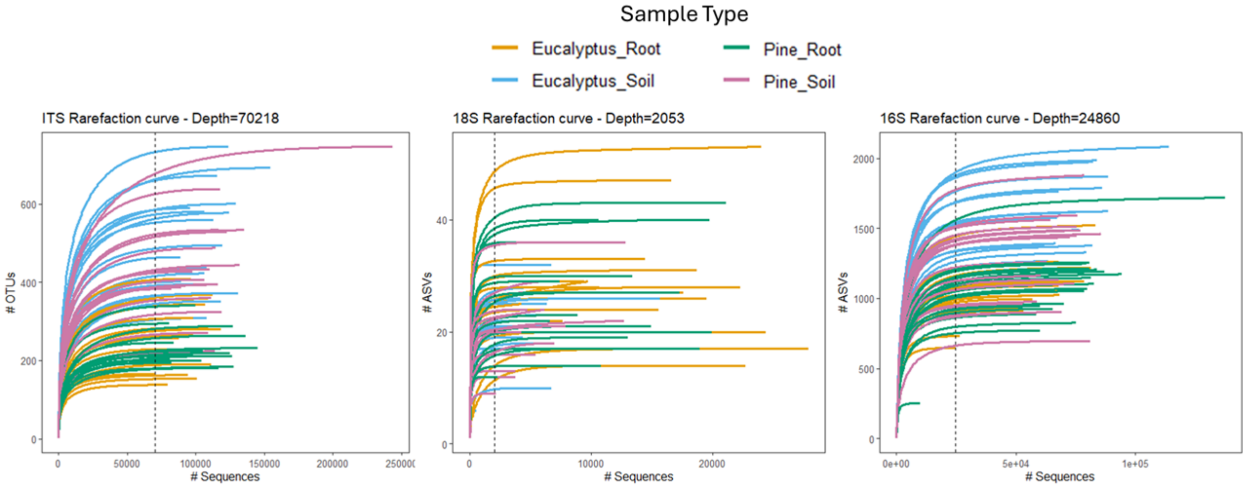

Fig. S6 Principal coordinate analysis (PCoA) assessing differences in arbuscular mycorrhizal fungal (AMF) community structure based on robust Aitchison distance. The first (PC1) and second (PC2) principal coordinate axes are shown for each sample type. *Pinus radiata* (Pine) samples are colored blue and *Eucalyptus racemosa* (Eucalypt) samples are colored yellow. Ellipses represent 95% confidence intervals. R^2 and P-values were calculated using permutational multivariate analysis of variance (PerMANOVA) tests. **a)** Root AMF (18S), **b)** Soil AMF (18S).

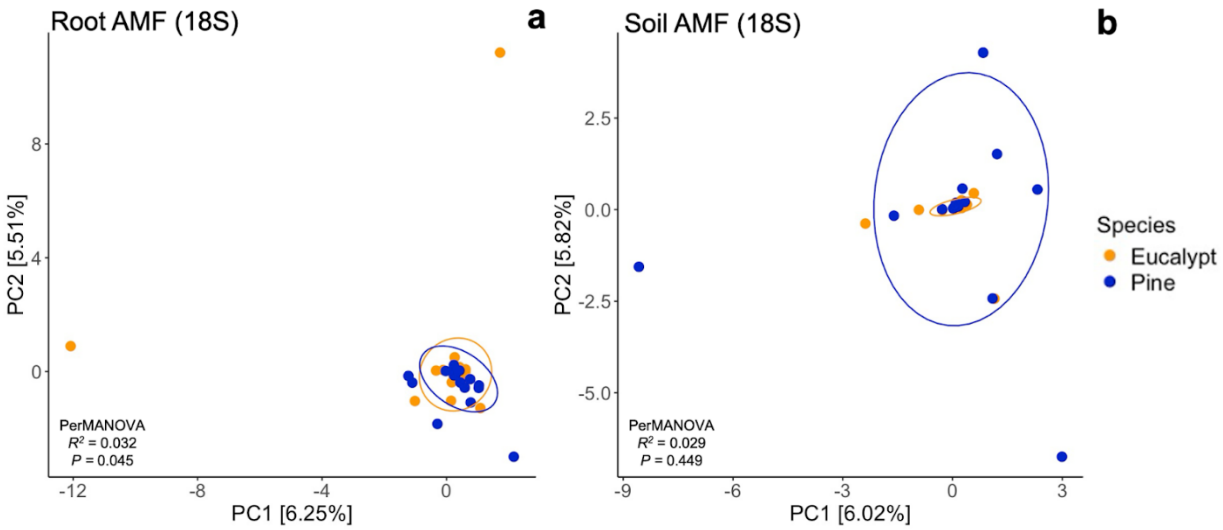

Fig. S7 Forest plot illustrating Cohen's *d* effect sizes for soil microbial property response variables associated with pines versus eucalypts. Negative effect sizes indicate variables favored under eucalypts, while positive effect sizes indicate variables favored under pines. Error bars show 95% confidence intervals.

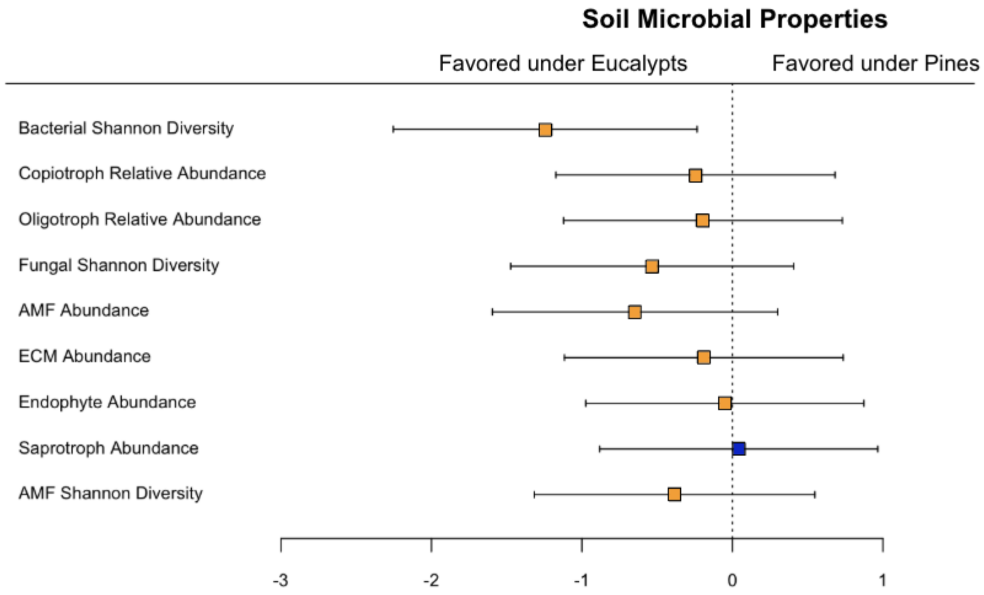

Fig. S8 Forest plot illustrating Cohen's *d* effect sizes for root microbial property response variables associated with pines versus eucalypts. Negative effect sizes indicate variables favored under eucalypts, while positive effect sizes indicate variables favored under pines. Error bars show 95% confidence intervals.

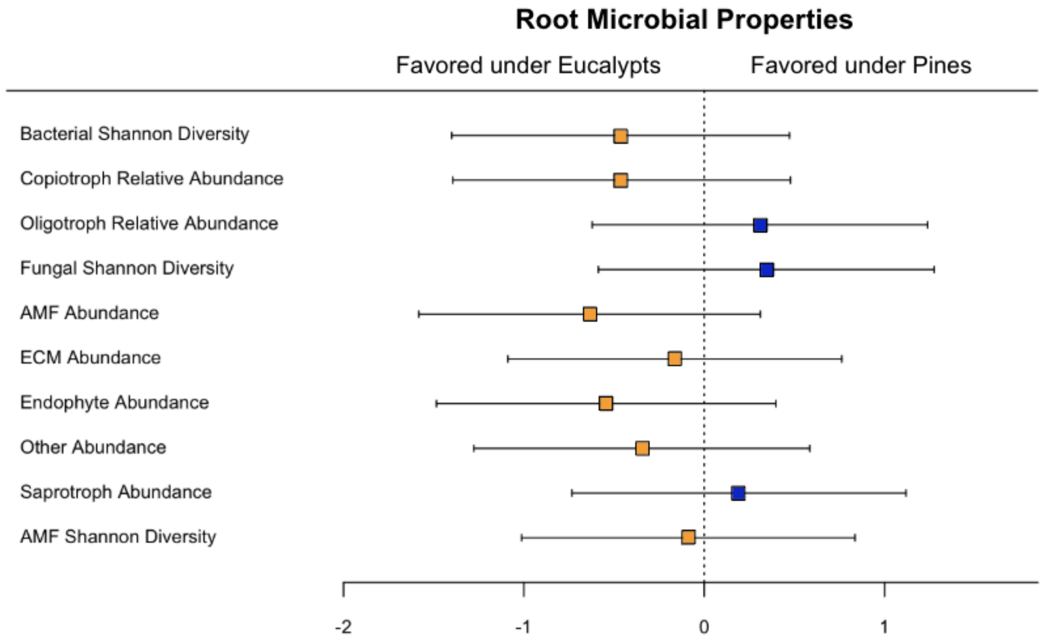

Fig. S9 An IQ-Tree 2 phylogeny generated from *Cantharellales* OTUs from this study (red tip labels), reference sequences (blue), and the *Sistotrema* OTUs from the Sapsford et al. (2022) study (green). Major clades and grades (paraphyletic groupings) are labelled. Numbers in parentheses indicate the number of OTUs from our study that are in each group. The provenance and isolation source for reference sequences is listed when available. We found one group of *Sistotrema* OTUs from our study that are likely ectomycorrhizal.

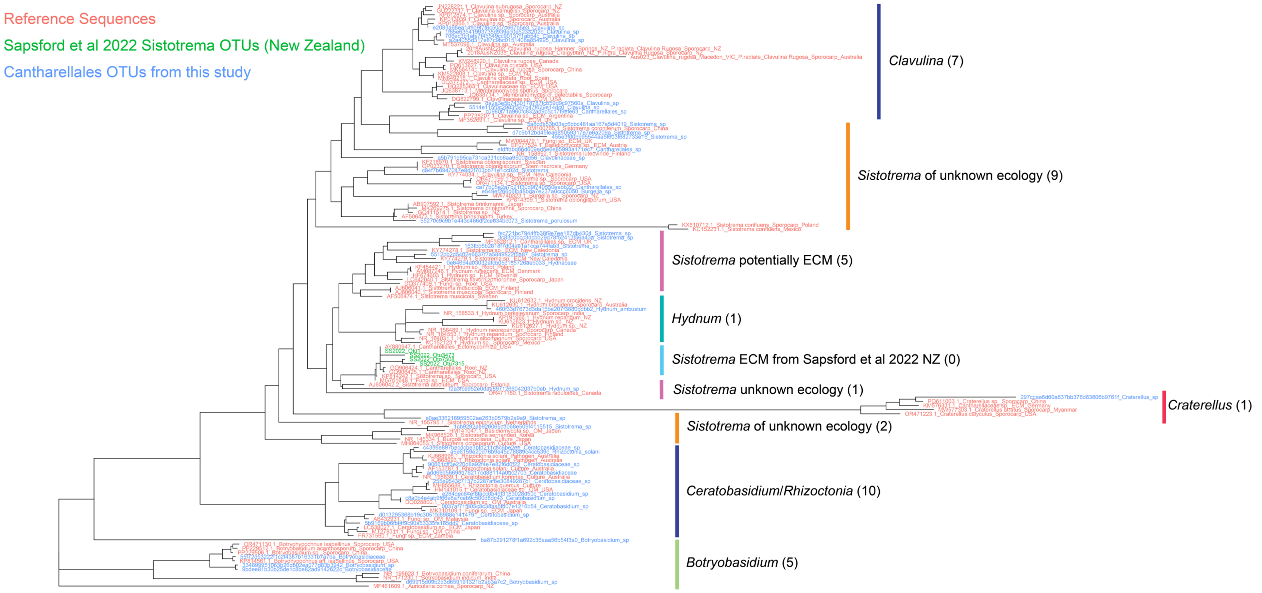

Fig. S10 Relative ITS2 sequence abundances of the introduced northern hemisphere fungal genera *Phialocephala*, *Rhizopogon*, and *Suillus* and "other ECM" which includes a mix of 267 other ectomycorrhizal OTUs. The "other" category includes fungi with other lifestyles (a mix of free-living saprotrophs, endophytes, plant pathogens, lichenized fungi, etc). Each bar along the x-axis represents an individual sample.

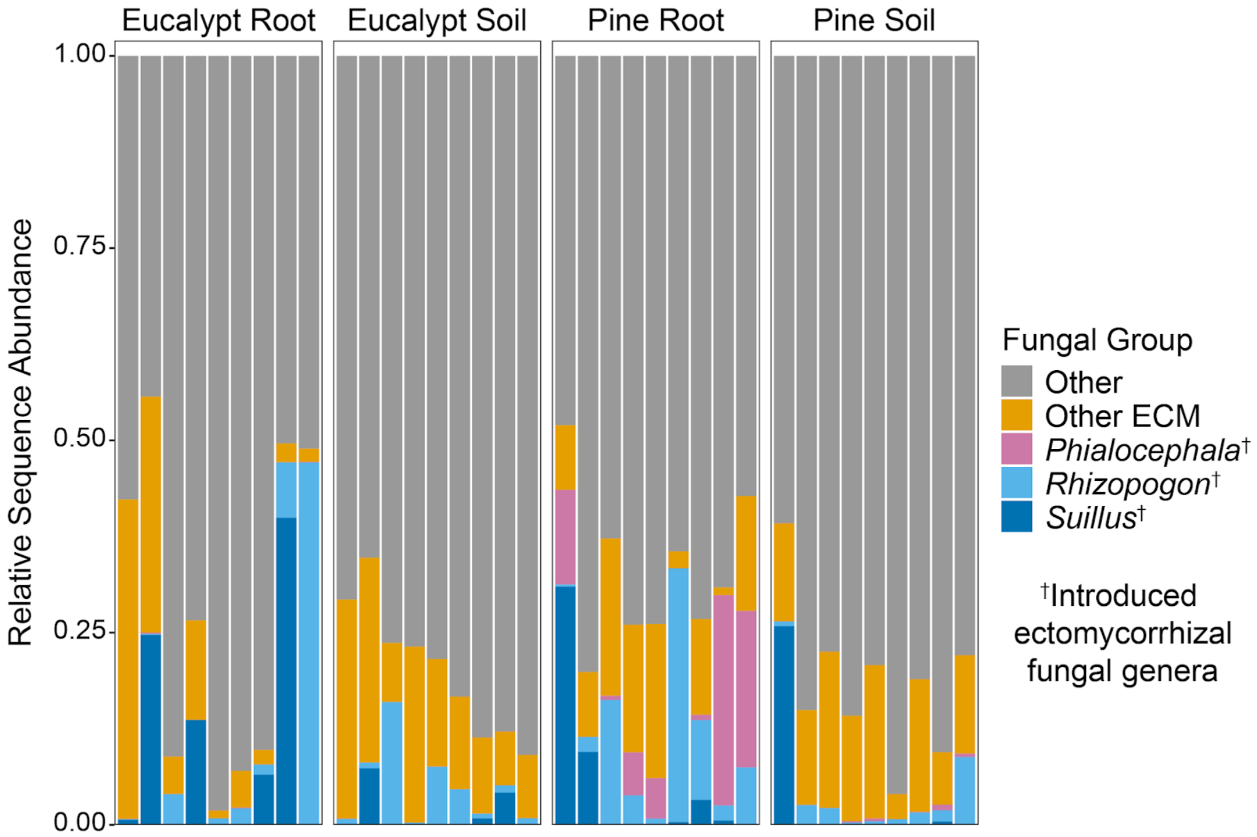

Fig. S11 Occurrence patterns of 12 OTUs of native Australian EMF in roots and soil on pines and eucalypts. None of the OTUs varied in abundance between pine and eucalypt soils or roots. OTU IDs and sequences for these OTUs are provided in the "ITS Taxonomic Assignments" tab of supporting Dataset S1. Boxes represent the interquartile range of the data, midpoint lines represents the median, whiskers represent data within 1.5 times the interquartile range, and points represent the values of each sample.

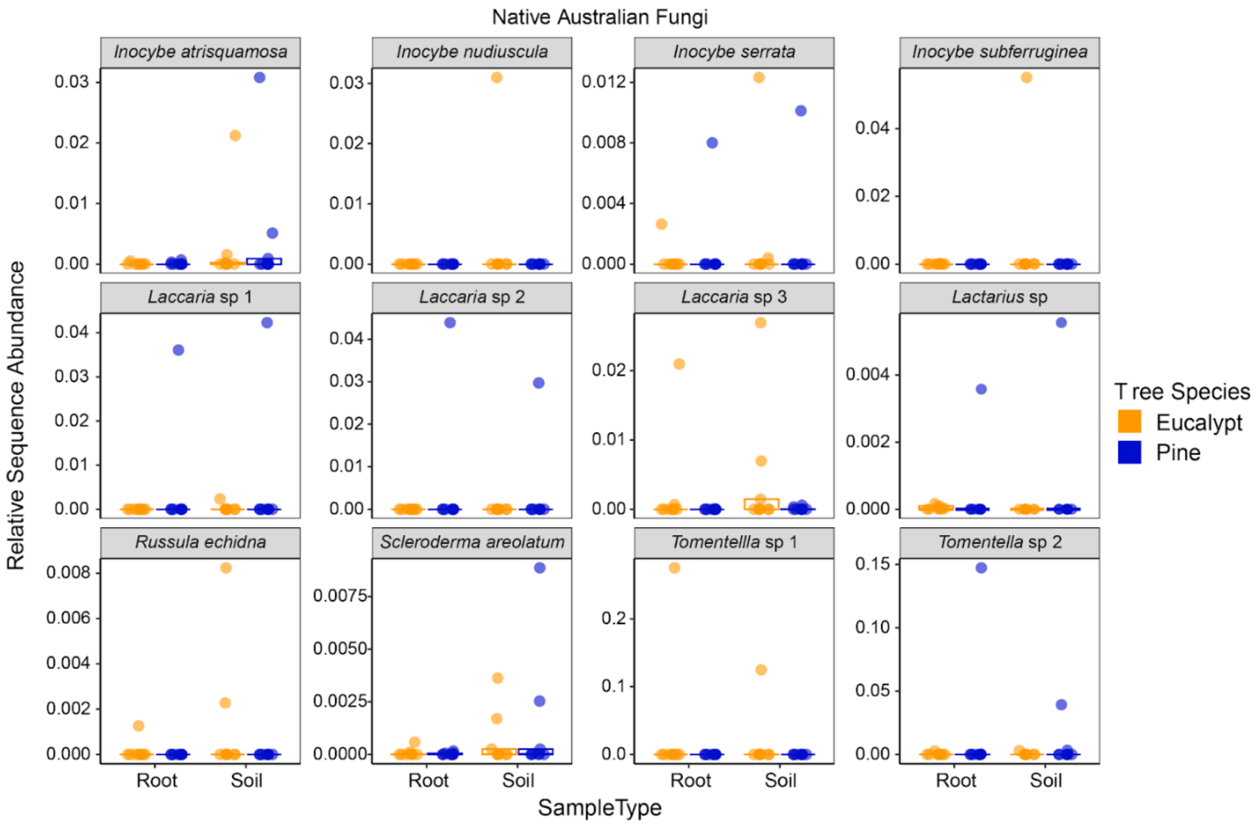

Fig. S12 Distance-based redundancy analysis (dbRDA) of soil microbial communities based on robust Aitchison distance of relative abundance at OTU level. The dbRDA axes represent the amount of variation explained by each axis under the constraining variables. Vectors represent environmental variables that were not significantly correlated with each other in a Pearson correlation. Bolded vectors represent environmental variables that were indicated as statistically significant indicators of soil microbial community composition. **a)** Soil bacterial (16S), **b)** Soil fungal (ITS), **c)** Soil AMF (18S).

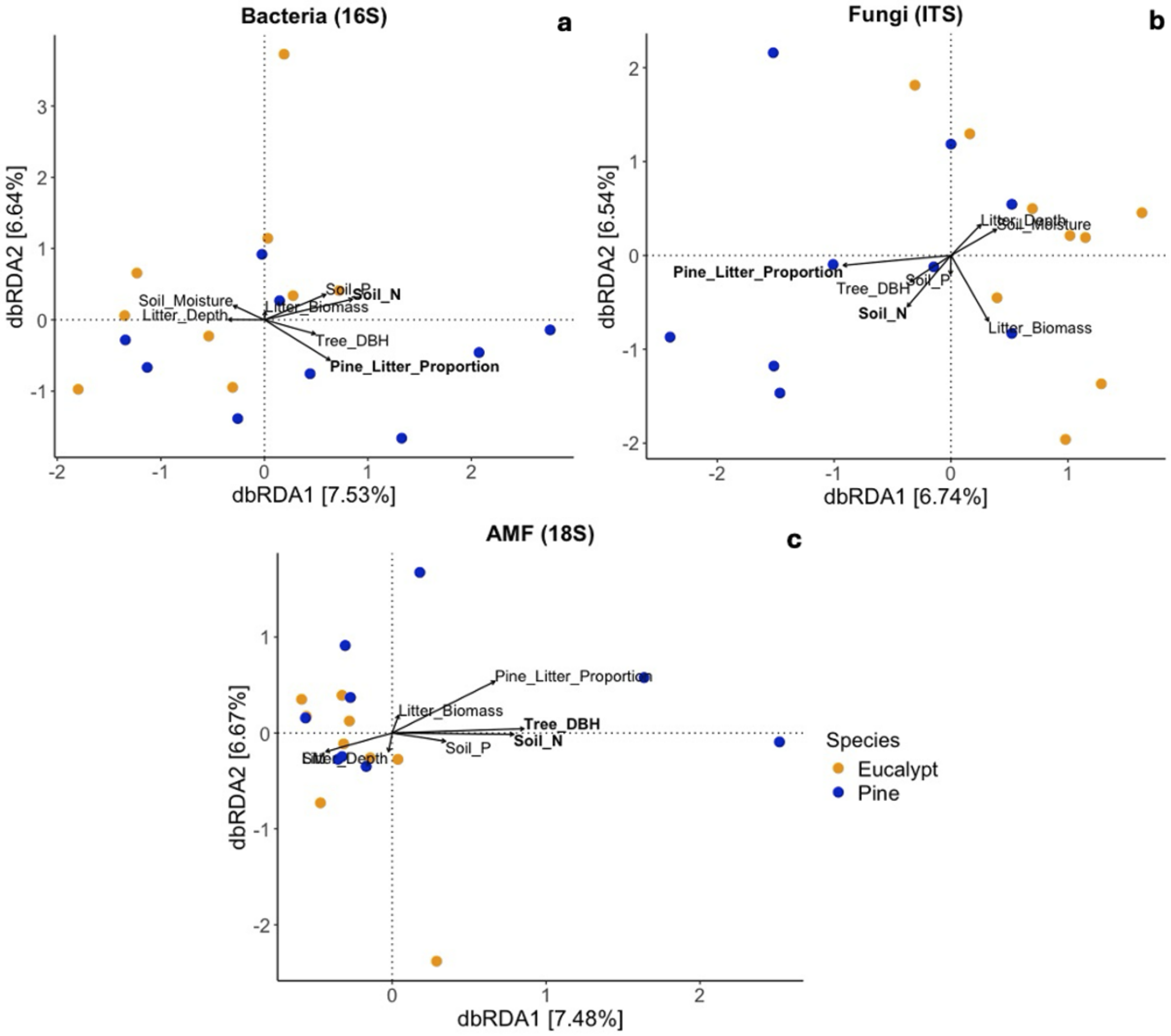

Methods S1 Methods description for ergosterol measurement, metabarcoding, bioinformatics, and spatial autocorrelation analysis

Ergosterol Extraction

We extracted ergosterol from ground litter (~150 mg) and soil (~1.0 g) subsamples in alcoholic KOH (0.8% KOH in HPLC-grade methanol) for 30 min at 80°C in tightly capped 50 ml polypropylene centrifuge tubes with plug seal cap (total extraction volume 10 ml). The resultant extract was partitioned into n-pentane and evaporated to dryness under a stream of N gas using a N-Evap 111. Ergosterol in dried samples was redissolved by sonication in 1 ml of HPLC-grade methanol, transferred to HPLC screw-capped autosampler vials, and stored at 20°C in the dark until quantified by a Shimadzu HPLC (Hendricks et al., 2016).

Sample Processing for metabarcoding

To obtain root samples for DNA metabarcoding, we hand-homogenized unsieved soil samples from the northern and southern plots of each tree. Using soil sieves and tap water, we washed away the soil and picked out fine roots of woody plants, examined them under a stereoscope for evidence of ectomycorrhizal colonization, then combined subsamples from the north and south plots into a single sample, before drying them at 52°C for 24 h. We stored dried root samples at room (ambient) temperature in the lab environment and during shipping and then froze them at -20°C following arrival at Duke University, where subsequent DNA extraction, amplification, and sequencing occurred. We ground dried root samples cryogenically with liquid nitrogen in a mortar and pestle.

DNA Extraction

We extracted DNA of roots and soil samples with the Qiagen PowerSoil Kit (QIAGEN 2023) following the protocol as written but reducing the amount of input material to 50 mg for roots and to 150 mg for soil and performed 1 min of bead beating in the lysis buffer with a BioSpec Products MiniBeadBeater-16 followed by a 10 min incubation at room temperature. Duplicate extractions were performed on root samples and single extractions on soil samples for a total of 72 DNA extractions.

We prepared amplicon sequencing libraries using degenerate primer mixes ITS3NGS and ITS4NGR to amplify ITS2 that cover certain fungal lineages with known primer mismatches (described in Cregger et al 2018; White et al. 1990; Tedersoo et al. 2014), primers 515F and 806R to amplify the V4 region of 16S, and primers WANDA and AML2 to amplify the SSU. These primer sets targeted total fungi, total prokaryotes, and Glomeromycota fungi (i.e., AMF) respectively. We carried out PCR reactions using a 3-step protocol, with the first step using unmodified primers to enrich target DNA, the second step using modified primers with an adapter region and frameshift section, and the third step using a universal forward primer and a sample-specific barcoded reverse primer. We incorporated an ITS peptide nucleic acid blocker to block the amplification of plant ITS2 sequences, and the pPNA and mPNA peptide nucleic acid blockers were used to block the amplification of plant plastid 16S and mitochondrial 16S, respectively. We used the Phusion Hot Start II High Fidelity polymerase for PCR reactions. Cycle conditions are listed in supporting Dataset S1. After the final PCR step, we checked amplicons on a 1% agarose gel and then cleaned up small fragments using the Omega Mag-Bind Bead Cleanup kit with a 0.9 ratio of beads:DNA. Following cleanup, we quantified amplicons using the Qubit HS dsDNA kit. We then pooled amplicons for the three loci at equal concentrations into a single library for sequencing, and the final library was again bead cleaned to adjust the DNA to the final concentration for sequencing. We performed sequencing at the Duke University Sequencing Core on an Illumina MiSeq using the V3 300 bp PE kit.

Microbial Community Bioinformatics and Annotation

We processed amplicon sequencing reads for 16S, ITS2, and 18S using QIIME2 (Bolyen et al., 2019), with methodology tailored to each locus, as described below. We used amplicon sequence variants (ASVs) as the unit of analysis for 16S and 18S data, while 97% operational taxonomic units (OTUs) were employed for ITS2 to account for the differing cntevolutionary rates between these loci (Estensmo et al., 2021). For consistency, we refer to all resulting data tables as “feature tables” and to ASVs and OTUs collectively as “taxa.”

We imported paired-end 16S rRNA sequences into QIIME2 (Bolyen et al., 2019), where we trimmed primers and adapter sequences using Cutadapt (Martin, 2011). We generated ASVs using DADA2 (Callahan et al., 2016) and taxonomically classified against the SILVA ribosomal database (Quast et al., 2012) using the classify-sklearn command in QIIME2 (Bokulich et al.,

2018; Pedregosa et al., 2011). We removed ASVs identified as mitochondrial or chloroplast sequences from downstream analysis. We assigned bacterial taxa as copiotrophs or oligotrophs using a database compiled from literature reviews and genomic pathway presence where we assigned guilds at the genus through phylum level (Averill et al. 2021).

Additionally, we merged ITS2 paired-end reads using PEAR (Zhang et al., 2014) and extracted the ITS2 region using ITSxpress (Rivers et al., 2018). We imported the processed sequences into QIIME2 (Bolyen et al., 2019) and denoised with DADA2 (Callahan et al., 2016). We clustered the resulting sequences into 97% OTUs using VSEARCH (Rognes et al., 2016). We assigned taxonomy to ITS2 representative sequences using the classify-sklearn command in QIIME2 (Bokulich et al., 2018; Pedregosa et al., 2011) with the UNITE fungal ITS database (Abarenkov et al., 2024). We conducted fungal guild annotation with a combination of FungalTraits and FunGuild (Nguyen et al., 2016; Põlme et al., 2020). We used a phylogenetic approach to identify ectomycorrhizal *Sistotrema* spp. because they are frequently highly abundant invasive fungi in southern hemisphere pine plantations, and they are highly polyphyletic which complicates identification (Sapsford et al. 2022). We made an ITS alignment with MAFFT (Katoh and Standley 2013) that contained the 4 *Sistotrema* OTUs from the (Sapsford et al. 2022) study, all Cantharellelas OTUs from our study, and 93 reference sequences from Cantharellelas sporocarps and ectomycorrhizal root tips that had high similarity to OTUs from our study. We constructed a phylogeny using IQ-TREE 2 (Minh et al. 2020) and visualized it using *ggtree* (Yu et al. 2017).

Furthermore, we obtained raw paired-end 18S sequences demultiplexed and carried out quality control in QIIME2 v.2023.5 (Bolyen et al., 2019). We trimmed primer regions and low-quality sequences and filtered chimeras using DADA2 (Callahan et al., 2016), resulting in 1,364 ASVs from 716,988 total sequences. We assigned taxonomy to representative sequences using the MaarjAM database (Öpik et al., 2010) and the feature-classifier plugin in QIIME2 (Bolyen et al., 2019; Bokulich et al., 2018). To identify ASVs belonging to the Glomeromycotina (the subphylum of arbuscular mycorrhizal fungi), we conducted sequence similarity searches at 95%, 90%, and 80% similarity thresholds while maintaining query coverage >90% and a BLAST e-value <1e-50, following methods similar to Kajihara et al. (2022). This yielded 622 ASVs at 95%, 658 ASVs at 90%, and 57 ASVs at 80% sequence similarity. Additionally, we compared remaining unassigned sequences to Glomeromycotina reference sequences from the NCBI

Nucleotide database at >80% sequence similarity, identifying six additional ASVs. We merged all ASVs into a single table using the QIIME2 feature-table plugin.

Finally, we imported feature tables and taxonomic annotations for all loci into RStudio for downstream analysis. Feature tables and taxonomic annotations are available in Supplemental File S2. We rarefied datasets to even sequencing depths of 2,053 for 18S, 24,860 for 16S, and 70,218 for ITS2. Rarefaction curves (shown in Supplemental Fig S5, below) demonstrate that these rarefaction depths successfully captured the vast majority of microbial diversity in almost all samples. After rarefaction, we pooled technical replicates from the same sample by summing their sequence abundances. This final dataset included 1,281 taxa for 18S, 18,775 taxa for 16S, and 4,159 taxa for ITS2. Generally, rarefied datasets were used for alpha diversity assessments and assessing associations between soil properties and soil microbial communities.

Spatial autocorrelation analyses

To ensure there was no spatial autocorrelation among samples, we ran a global Moran's I test using the *spdep* package (Pebesma & Bivand, 2023) on all environmental variables that differed significantly between pines and eucalypts (soil and litter properties, microbial diversity metrics, and Biolog substrate activities), the top 5 bacterial and fungal genera enriched under eucalypt and pine soils and roots, and the first PCoA axis of microbial community composition for each gene region (16S, ITS, 18S) and sample type (roots and soils). No autocorrelation was detected for any environmental variables or microbial composition PCoA axes, and only 7 out of 40 microbial genera examined had significant spatial autocorrelation (supporting Dataset S1). We also ran linear regressions to ensure latitude and longitude did not have an effect in models testing the effects of tree species and DBH on microbial variables. Following the same linear regression methods as described in the main text, we constructed linear regressions using the microbial PCoA axes and enriched genera as response variables, and latitude, longitude, tree species, tree DBH, and tree species*DBH as predictors. Latitude and longitude were not significant predictors for any PCoA axes, and both were significant only for the abundance of one fungal genus in soils out of the 40 genera we tested (*Leohumicola,* Supporting Dataset S1). Thus, no samples were omitted from analyses due to spatial autocorrelation.

<u>**References for Methods S1**</u>

Abarenkov K, Nilsson RH, Larsson K-H, Taylor AF, May TW, Frøslev TG, Pawlowska J, Lindahl B, Põldmaa K, Truong C. **2024**. The UNITE database for molecular identification and taxonomic communication of fungi and other eukaryotes: sequences, taxa and classifications reconsidered. *Nucleic Acids Research* **52**: D791–D797.

Averill C, Werbin ZR, Atherton KF, Bhatnagar JM, Dietze MC. **2021**. Soil microbiome predictability increases with spatial and taxonomic scale. *Nature Ecology & Evolution* **5**: 747–756.

Bokulich NA, Kaehler BD, Rideout JR, Dillon M, Bolyen E, Knight R, Huttley GA, Gregory Caporaso J. **2018**. Optimizing taxonomic classification of marker-gene amplicon sequences with QIIME 2's q2-feature-classifier plugin. *Microbiome* **6**: 1–17.

Bolyen E, Rideout JR, Dillon MR, Bokulich NA, Abnet CC, Al-Ghalith GA, Alexander H, Alm EJ, Arumugam M, Asnicar F. **2019**. Reproducible, interactive, scalable and extensible microbiome data science using QIIME 2. *Nature biotechnology* **37**: 852–857.

Callahan BJ, McMurdie PJ, Rosen MJ, Han AW, Johnson AJA, Holmes SP. **2016**. DADA2: High-resolution sample inference from Illumina amplicon data. *Nature methods* **13**: 581–583.

Cregger MA, Veach AM, Yang ZK, Crouch MJ, Vilgalys R, Tuskan GA, Schadt CW. **2018**. The Populus holobiont: dissecting the effects of plant niches and genotype on the microbiome. *Microbiome* **6**: 31.

Estensmo ELF, Maurice S, Morgado L, Martin-Sanchez PM, Skrede I, Kauserud H. **2021**. The influence of intraspecific sequence variation during DNA metabarcoding: A case study of eleven fungal species. *Molecular Ecology Resources* **21**: 1141–1148.

Hendricks JJ, Mitchell RJ, Kuehn KA, Pecot SD. **2016**. Ectomycorrhizal fungal mycelia turnover in a longleaf pine forest. *New Phytologist* **209**: 1693–1704.

Hoeksema JD, Averill C, Bhatnagar JM, Brzostek E, Buscardo E, Chen K-H, Liao H-L, Nagy L, Policelli N, Ridgeway J, *et al.* **2020**. Ectomycorrhizal Plant-Fungal Co-invasions as Natural Experiments for Connecting Plant and Fungal Traits to Their Ecosystem Consequences. *Frontiers in Forests and Global Change* **3**.

Kajihara KT, Egan CP, Swift SOI, Wall CB, Muir CD, Hynson NA. **2022**. Core arbuscular mycorrhizal fungi are predicted by their high abundance–occupancy relationship while host-specific taxa are rare and geographically structured. *New Phytologist* **234**: 1464–1476.

Katoh K, Standley DM. 2013. MAFFT multiple sequence alignment software version 7: improvements in performance and usability. *Molecular Biology and Evolution* **30**: 772-780.

Martin M. **2011**. Cutadapt removes adapter sequences from high-throughput sequencing reads. *EMBnet. journal* **17**: 10–12.

Minh BQ, Schmidt HA, Chernomor O, Schrempf D, Woodhams MD, Von Haeseler A, Lanfear, R. 2020. IQ-TREE 2: new models and efficient methods for phylogenetic inference in the genomic era. *Molecular Biology and Evolution* **37**: 1530-1534.

Nguyen NH, Song Z, Bates ST, Branco S, Tedersoo L, Menke J, Schilling JS, Kennedy PG. **2016**. FUNGuild: An open annotation tool for parsing fungal community datasets by ecological guild. *Fungal Ecology* **20**: 241–248.

Öpik M, Vanatoa A, Vanatoa E, Moora M, Davison J, Kalwij JM, Reier Ü, Zobel M. **2010**. The online database MaarjAM reveals global and ecosystemic distribution patterns in arbuscular mycorrhizal fungi (Glomeromycota). *New Phytologist* **188**: 223–241.

Pebesma E, Bivand R. **2023**. *Spatial Data Science: With Applications in R*. New York: Chapman and Hall/CRC.

Pedregosa F, Varoquaux G, Gramfort A, Michel V, Thirion B, Grisel O, Blondel M, Prettenhofer P, Weiss R, Dubourg V. **2011**. Scikit-learn: Machine learning in Python. *the Journal of machine Learning research* **12**: 2825–2830.

Põlme S, Abarenkov K, Henrik Nilsson R, Lindahl BD, Clemmensen KE, Kauserud H, Nguyen N, Kjøller R, Bates ST, Baldrian P, *et al.* 2020. FungalTraits: a user-friendly traits database of fungi and fungus-like stramenopiles. *Fungal Diversity* **105**: 1–16.

Quast C, Pruesse E, Yilmaz P, Gerken J, Schweer T, Yarza P, Peplies J, Glöckner FO. **2012**. The SILVA ribosomal RNA gene database project: improved data processing and web-based tools. *Nucleic acids research* **41**: D590–D596.

Rivers AR, Weber KC, Gardner TG, Liu S, Armstrong SD. **2018**. ITSxpress: Software to rapidly trim internally transcribed spacer sequences with quality scores for marker gene analysis. *F1000Research* **7**.

Rognes T, Flouri T, Nichols B, Quince C, Mahé F. **2016**. VSEARCH: a versatile open source tool for metagenomics. *PeerJ* **4**: e2584.

Sapsford SJ, Wakelin A, Peltzer DA, Dickie IA. **2022**. Pine invasion drives loss of soil fungal diversity. *Biological Invasions* **24**: 401–414.

Tedersoo L, Bahram M, Põlme S, Kõljalg U, Yorou NS, Wijesundera R, Ruiz LV, Vasco-Palacios AM, Thu PQ, Suija A, *et al.* 2014. Global diversity and geography of soil fungi. *Science* **346**: 1256688.

White T, Bruns T, Lee S, Taylor J, Innis M, Gelfand D, Sninsky J. **1990**. Amplification and Direct Sequencing of Fungal Ribosomal RNA Genes for Phylogenetics. In: Pcr Protocols: a Guide to Methods and Applications,. 315–322.

Yu G, Smith DK, Zhu H, Guan Y, Lam TTY. (2017). GGTREE: an R package for visualization and annotation of phylogenetic trees with their covariates and other associated data. *Methods in Ecology and Evolution* **8**: 28-36.

Zhang J, Kobert K, Flouri T, Stamatakis A. **2014**. PEAR: a fast and accurate Illumina Paired-End reAd mergeR. *Bioinformatics* **30**: 614–620.
